# Molecular Modification of Queen Bee Acid and 10-Hydroxydecanoic Acid with Specific Tripeptides: Rational Design, Organic Synthesis, and Assessment for Prohealing and Antimicrobial Hydrogel Properties

**DOI:** 10.3390/molecules30030615

**Published:** 2025-01-30

**Authors:** Song Hong, Sachin B. Baravkar, Yan Lu, Abdul-Razak Masoud, Qi Zhao, Weilie Zhou

**Affiliations:** 1 Neuroscience Center of Excellence, School of Medicine, Louisiana State University Health, New Orleans, LA 70112, USAamasou@lsuhsc.edu (A.-R.M.); 2 Department of Ophthalmology, School of Medicine, Louisiana State University Health, New Orleans, LA 70112, USA; 3 NMR Laboratory, Department of Chemistry, Tulane University, New Orleans, LA 70115, USA; qzhao@tulane.edu; 4 Department of Physics and AMRI, University of New Orleans, New Orleans, LA 70148, USA

**Keywords:** royal jelly and medical grade honey, hydroxylated fatty acid, wound healing, infection, viscoelastic moduli, shear-thinning

## Abstract

Royal jelly and medical grade honey are traditionally used in treating wounds and infections, although their effectiveness is often variable and insufficient. To overcome their limitations, we created novel amphiphiles by modifying the main reparative and antimicrobial components, queen bee acid (hda) and 10-hydroxyl-decanoic acid (hdaa), through peptide bonding with specific tripeptides. Our molecular design incorporated amphiphile targets as being biocompatible in wound healing, biodegradable, non-toxic, hydrogelable, prohealing, and antimicrobial. The amphiphilic molecules were designed in a hda(hdaa)-aa1-aa2-aa3 structural model with rational selection criteria for each moiety, prepared via Rink/Fmoc-tBu-based solid-phase peptide synthesis, and structurally verified by NMR and LC–MS/MS. We tested several amphiphiles among those containing moieties of hda or hdaa and isoleucine–leucine–aspartate (ILD-amidated) or IL-lysine (ILK-NH_2_). These tests were conducted to evaluate their prohealing and antimicrobial hydrogel properties. Our observation of their hydrogelation and hydrogel-rheology showed that they can form hydrogels with stable elastic moduli and injectable shear-thinning properties, which are suitable for cell and tissue repair and regeneration. Our disc-diffusion assay demonstrated that hdaa-ILK-NH_2_ markedly inhibited *Staphylococcus aureus*. Future research is needed to comprehensively evaluate the prohealing and antimicrobial properties of these novel molecules modified from hda and hdaa with tripeptides.

## 1. Introduction

Queen bee acid (10-hydroxy-2-decenoic acid or hda) and 10-hydroxydecanoic acid (hdaa) are the bioactive components of royal jelly and medical grade honey [1,2,3,4,5,6]. Medical grade honey (MGH) and royal jelly (RJ) are used to heal various wounds, especially infected wounds, pressure ulcers (bedsores), diabetic ulcers, and/or venous or arterial ulcers [1,7,8,9,10,11,12,13,14,15,16]. Mitogen-Activated Protein Kinase (MAPK) signaling can be activated by hda [5]. Reports have shown that hda reduces inflammatory cytokine and signaling, exerts bactericidal activity against infections in gastrointestinal tract [17,18], promotes smooth muscle cell survival against hydroxyl radical-induced injury [19], prevents photoaging damage by enhancing collagen production in human dermal fibroblasts [20], and promotes neurogenesis by neural stem cells [21]. Hda alleviated blood–brain barrier damage via activating the MAPK/PI3K/AKT pathway [22], increases longevity and TOR signaling in *C. elegans*, and fortifies immunity [23]. The mechanism for hda actions also involves reducing IκB-ζ expression [24], inhibiting the TNF-α/NF-κB axis and NLRP3 inflammasome-IL-1β pathway, increasing FOXO1-activation of autophagy [23], and down-regulating MMPs [25]. Hda and hdaa contribute to the key pharmacological effects of royal jolly, including anti-inflammatory, wound healing, anti-oxidant, anti-bacterial, and insulin-like properties [1,5,26,27,28]. Despite their potential, the effectiveness of hda, hdaa, MGH, or RJ are still insufficient to meet the clinical demand for healing infected or chronic wounds [1,7,8,9,10,11,12,13,14,15,16]. Furthermore, the efficacies of MGH and RJ are inconsistent due to their compositions being dependent on honeybees and flowers, and this varies significantly. This variability makes it challenging to enforce quality-control and regulation [1], posing a high risk to patients. Since they are products of flowers and bees, MGH/RJ can trigger allergic reactions [29,30]. Such reactions can be avoided, though, if the allergens are removed from the formulation. Gels and other wound dressings [1,31,32] and nanomaterials [1,33,34] have been used to provide slow release of MGH/RJ, but still do not resolve these weaknesses.

Wound healing is a crucial physiological process that allows the body to repair tissue damage from injuries or surgeries—thus preventing infection, maintaining tissue integrity, and enabling recovery from various medical procedures. Impeded wound healing potentially results in serious complications, like chronic ulcers, infections, and tissue loss, significantly impairing a patient’s quality of life and overall health [35,36,37,38]. The field of regenerative hydrogels is emerging and has been explored for ideal treatment of wounds. Hydrogels can hold large amounts of biological fluid, mimic the 3D structure of tissue extracellular matrix to scaffold tissue repair and regeneration [39,40,41,42,43,44,45] and barricade wounds against the external environment. They are semi-permeable to H_2_O vapor and O_2_ and CO_2_ exchange. Hydrogel flexibility and ability to conform to wound contours offer optimal tissue contact, which as a result optimizes the healing process. Hydrogels can be engineered to release their therapeutics at the optimal rate and concentration, or in response to specific environmental or pathological change. Hydrogels can also disrupt microbial biofilms, prevent biofilm re-formation, and make bacteria more susceptible to treatment and the host immune system [46]. Additionally, hydrogel dressing can be removed without the pain typically associated with changing non-hydrogel dressings [47], like gauzes, plasters, and bandages. Therefore, we incorporate hydrogel functions into our therapeutic development.

*Staphylococcus aureus*, a major bacterium in wound infections, often develops antimicrobial resistance (AMR), leading to life-threatening conditions [48]. Methicillin-resistant *S. aureus* (MRSA) causes worse clinical outcomes. *S. aureus* in biofilms exhibits increased virulence and AMR, which makes antimicrobials less effective, resulting in infected wounds [49,50,51]. There is a lack of effective therapies regarding the AMR of *S. aureus* for non-healing infected wounds [52,53,54], particularly among older adults, underscoring the need for innovative therapeutics. Moreover, *S. aureus* can cause a broad range of diseases in humans and/or animals, including pneumonia, endocarditis, osteomyelitis, mastitis, toxic shock syndrome, cellulitis, and impetigo [55,56]. In the United States alone, the economic burden of community-associated MRSA (CA-MRSA) ranges from $478 million to $2.2 billion annually for third-party payers and from $1.4 billion to $13.8 billion for society [57].

This report presents initial approaches to the development of therapeutic molecules that have the potential to enhance the medicinal functions of MGH/RJ and their reparative components, hda and hdaa, and to overcome the limitations of these natural products. We performed the molecular modification of hda and hdaa with specific tripeptides through rational design and solid phase organic synthesis, aiming to create prohealing antimicrobial amphiphiles. Our goal is to engineer these novel amphiphiles to be biocompatible in wound healing, biodegradable, non-toxic, hydrogelable in the wound niche, prohealing, and antimicrobial. Additionally, we generated novel molecules by replacing hda or hdaa in these amphiphiles with representative long linear carbon carboxylic acids, including 3-hydroxyl-decanoic acid (a minor RJ component), 12-hydroxyl dodecanoic acid, and 12-hydroxyl octadecanoic acid, for comparison and to explore effects of the hydroxyl fatty acid structure on the amphiphile functions. We then assessed the self-assembling hydrogelability and anti-*S aureus* property of selected molecules that we engineered.

## 2. Results and Discussion

### 2.1. Rational Design and Solid Phase Organic Synthesis of Novel Amphiphiles Covalently Modified from hda, hdaa, or Other Hydroxy Fatty Acids with Specific Tripeptides

Amphiphilic molecular structures were targeted for our covalent modification of hda and hdaa using specific peptides. Appropriately designed amphiphiles can form prohealing hydrogels under wound-compatible conditions [58,59,60,61,62,63,64,65]. Amphiphiles possess a hydrophilic head and a hydrophobic tail. Their ability to form hydrogels depends on the balance between these regions. This balance facilitates self-assembly into complex structures (e.g., fibrils or networks) through non-covalent interactions, such as hydrogen bonding, electrostatic forces, and van der Waals interactions. This self-assembly results in a hydrogel-like network that can retain large amounts of water. Factors such as the length and structure of the hydrophobic tail, as well as the polarity of the hydrophilic head, play a crucial role in this process [66,67,68,69]. Certain amphiphiles target bacterial cell membranes, disrupting or compromising their integrity while sparing mammalian cell membranes. These include cationic amphiphiles inspired by natural antimicrobial peptides [70,71,72]. Bacterial membranes are generally more negatively charged, which allows positively charged amphiphiles to bind strongly and disrupt them. In contrast, mammalian membranes have a more neutral charge, reducing interaction and potential damage [73,74,75]. When an amphiphile interacts with a bacterial membrane, its hydrophobic region inserts into the lipid bilayer, while the hydrophilic region interacts with the aqueous environment. This interaction disrupts the membrane structure, leading to cell leakage and death. Depending on the structure, amphiphiles can also form pores in the bacterial membrane, allowing the leakage of essential cellular components [76]. The amphiphile structure dependent balance between the hydrophobic and hydrophilic regions of an amphiphile significantly affects its selectivity between bacterial membranes and mammalian membranes [72]. The arrangement of positive charges on the amphiphile molecule can also influence its interaction with different membranes [70].

We linked hda or hdaa via peptide bond by solid phase organic synthesis to a rationally designed tripeptide in order to create the gelable Janus amphiphile, suitable in promoting healing and inhibiting infection [77]. This amphiphile has the hda or hdaa moiety acting as a suitable hydrophobic tail and the tripeptide domain providing the appropriate hydrophilic head and aqueous solubility, so linkage to hda/hdaa, consequently, is able to self-assemble into a prohealing and antimicrobial hydrogel. Based on knowledge of hydrogelable covalently bonded conjugates of fatty acid and tripeptides that we developed recently [78,79], we selected a tripeptide moiety. Longer peptides contain more peptide bonds, which provide additional sites for enzymes that break down these bonds during biodegradation [80,81], and cost more in preparation. Dipeptide or mono-amino acid moieties generally have lower intermolecular interaction (hydrogen bonds and hydrophobic force) than their tripeptide counterparts. Therefore, they were not selected for this pilot study [82,83,84,85]. However, it would be worth exploring dipeptides or mono-amino acids as the moiety in the future to understand the effect of the length of peptide sequence on hydrogelation of hda(hdaa)-peptide bonded molecules. Another advantage of using specific tripeptides is that they are biocompatible and biodegradable. They can be broken down by tissue enzymes into individual nutrient amino acids [86].

Based on this rationale, we designed an amphiphilic molecular structural model linking a hydroxyl fatty acid (hfa) and a specific tripeptide via a covalent bond “–”, namely hfa–amino acid1–amino acid2–amino acid3 or hfa–aa1–aa2–aa3. The hfa includes hda, hdaa, 3-hydroxy-decanoic acid (a minor RJ component), 12-hydroxyl dodecanoic acid, 12-hydroxyl octadecanoic acid, or other hydroxylated fatty acids. The aa1 includes a hydrophobic amino acid that can enhance the hydrophobicity of our targeted amphiphile, such as alanine (Ala or A), valine (Val or V), isoleucine (Ile or I), leucine (Leu or L), methionine (Met or M), phenylalanine (Phe or F), tyrosine (Tyr or Y), or tryptophan (Trp or W). The aa2 can facilitate the hydrophobicity or act as a linker. These can be hydrophobic, including A, V, I, L, M, F, Y, or W. The aa2 can also facilitate hydrophilicity and provide linkage, so it can be polar uncharged, such as serine (Ser or S), threonine (Thr or T), asparagine (Asn or N), glutamine (Gln or Q); have other case side chains, such as glycine (gly or G), cysteine (Cys or C), proline (Pro or P), or selenocysteine (Sec or U); or be charged, such as arginine (Arg or R), lysine (Lys or K), aspartic acid (Asp or D), and glutamic acid (Glu or E). The aa3 at the C-terminus of the tripeptide provides the appropriate hydrophilic head and aqueous solubility for our targeted amphiphile. This includes an amino acid with an electrically charged side-chain that can function as the hydrophilic head, such as R, K, D, or E. At pathophysiological pH (~7.4), R or K is positively charged due to its side chain containing a guanidinium (pKa ~12.5) or alkyl amino group (pKa ~10.7) [87]. R or K side chains remain protonated and positively charged while its terminus carboxyl is amidated, and α-amino group is used in the peptide bond linking to aa2, providing the hydrophilic head and aqueous solubility for gelation. In contrast, D or E contains a net negative charge because its carboxyl side chain loses its proton (with pKa ~3.9 for D or ~4.1 for E), while the terminus carboxyl is free or amidated, and α-amino group is used in the peptide bond linked to aa2, providing the hydrophilic head and aqueous solubility for gelation [87]. Histidine (His or H) can also be used as aa3 due to its imidazole side chain (pKa 6.0) being protonated and hydrophilic at pH < 5, a wound condition during treatment [88,89]. The terminus carboxyl is amidated and α-amino group is used in the peptide bond linking to aa2, providing the hydrophilic head and aqueous solubility for gelation.

This hfa-aa1–aa2–aa3 molecular structural model is modified from our recently developed amphiphilic molecular model fatty acid Cn -aa1–aa2–aspartic acid (D) tripeptide, which has been partially presented in our recent article and associated provisional patent application, where the carbon chain length is n for the fatty acid Cn [78,79]. The hydroxyl of hfa reduces its hydrophobicity compared to the Cn with the same carbon chain length, whereas this hydroxyl can donate hydrogen for hydrogen bonding that could facilitate hydrogelation.

**Scheme 1:**
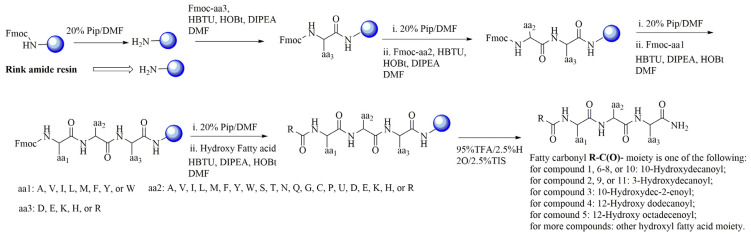
General strategy for Fmoc/tBu-based solid-phase peptide synthesis (SPPS) of a novel amphiphile from its moiety via peptide bond linkage under the molecular sequence model as hydroxyl fatty acid-amino acid 1-amino acid 2-amino acid 3 (hfa-aa1-aa2-aa3).

The synthesis of these hfa-aa1–aa2–aa3 amphiphiles was carried out through standard Fmoc-based solid phase peptide synthesis (SPPS) using Rink-amide-resin (Scheme 1). The representative novel amphiphiles generated from the modification of hda, hdaa, and several other types of hfa by this approach are presented in Table 1. More of these novel amphiphiles are presented in the Supplementary Materials.

### 2.2. NMR Data Verified the Structures of the Molecules Synthesized

The NMR spectra for compounds **1**–**6** provided chemical shifts δ and coupling constants J that verify their designed molecular structures.

Compound **1** (hdaa-ILD-NH_2_). Its structure was verified by our NMR data as follows: ^1^H NMR (400 MHz, DMSO-d_6_) (Figure S1): δ 12.23 (brs, 1H), 7.96 (t, J = 8.4 Hz, 2H), 7.87 (d, J = 10.4 Hz, 2H), 7.09 (d, J = 6.1 Hz, 2H), 4.41 (t, J = 7.1 Hz, 1H), 4.37 (t, J = 8.4 Hz, 1H), 4.24 (q, J = 10.0 Hz, 1H), 4.10 (t, J = 10.0 Hz, 1H), 2.65 (q, J = 6.1 Hz, 1H), 2.54 (d, J = 6.1 Hz, 1H), 2.14–2.10 (m, 2H), 1.70–1.66 (m, 2H), 1.59–1.51 (m, 2H), 1.47–1.39 (m, 5H), 1.29–1.24 (m, 12H), 0.87 (d, J = 8.2 Hz, 3H), 0.81 (s, J = 8.2 Hz, 9H); ^13^C NMR (100 MHz, DMSO-d_6_) (Figures S2 and S3): δ 172.4, 172.2, 171.8, 171.6, 171.4, 68.5, 63.6, 60.7, 57.0, 51.2, 49.4, 40.2, 35.8, 35.6, 33.5, 32.5, 29.0, 28.8, 28.7, 28.6, 28.5, 28.4, 28.3, 28.1, 27.4, 25.5, 25.3, 24.9, 23.0, 21.4, 15.4, 10.7.

Compound **2** (3-hydroxyl decanoic acid-ILD-NH_2_) (Figures S4–S6):. ^1^H NMR (400 MHz, DMSO-d6) (Figure S4): δ 12.18 (brs, 1H), 8.13–8.07 (m, 1H), 8.01–7.88 (m, 2H), 7.10–7.03 (m, 2H), 5.44–5.34 (m, 1H), 4.66 (m, 1H), 4.44–4.39 (m, 1H), 4.28–4.10 (m, 2H), 3.76 (brs 1H), 2.69–2.55 (m, 2H), 2.28–2.19 (m, 1H), 2.09 (s, 2H), 1.66–1.56 (m, 2H), 1.46–1.42 (m, 4H), 2.09 (s, 1H), 1.66–1.56 (m, 2H), 1.46–1.42 (m, 4H), 1.28 (brs, 10H), 0.88 (brs, 3H), 0.82 (m, 9H).

Compound **3** (hda-ILD-NH_2_). Its structure was verified by NMR data as follows. ^1^H NMR (400 MHz, DMSO-d6) (Figure S7): δ 12.18 (brs, 1H), 8.06 (d, J = 8.5 Hz, 2H), 7.99 (d, J = 8.5 Hz, 1H), 7.08 (d, J = 14.0 Hz, 1H), 6.67–6.60 (m, 1H), 6.08 (d, J = 16.0 Hz, 1H), 4.43–4.36 (m, 2H), 4.25–4.17 (m, 2H), 3.38 (t, J = 13.0 Hz, 1H), 2.68–2.63 (m, 1H), 2.55 (d, J = 7.5 Hz, 1H), 2.15–2.10 (m, 2H), 1.76–1.67 (m, 1H), 1.62–1.52 (m, 1H), 1.48–1.39 (m, 6H), 1.31–1.26 (m, 8H), 0.87 (d, J = 8.5 Hz, 3H), and 0.81 (d, J = 8.5 Hz, 9H); ^13^C NMR (100 MHz, DMSO-d6) (Figures S8 and S9): δ 172.3, 171.8, 171.7, 171.3, 165.2, 143.0, 124.2, 68.5, 60.6, 57.2, 51.3, 49.5, 36.2, 35.8, 32.5, 31.2, 28.7, 28.6, 28.4, 28.2, 27.7, 27.6, 27.3, 25.4, 24.9, 24.5, 24.0, 23.0, 21.4, 15.3, 10.9.

Compound **4** (12-hydroxyl dodecanoic acid-ILD-NH_2_). Its structure was verified by NMR data as follows. ^1^H NMR (400 MHz, DMSO-d6) (Figure S10): δ 12.26 (brs, 1H), 7.96 (t, J = 8.4 Hz, 2H), 7.88 (d, J = 7.2 Hz, 1H), 7.10 (d, J = 5.1 Hz, 2H), 4.42–4.36 (m, 2H), 4.24 (q, J = 7.2 Hz, 1H), 3.99 (t, J = 8.3 Hz, 1H), 3.34 (brs, 2H), 2.67–2.61 (m, 1H), 2.55 (d, J = 8.0 Hz, 1H), 2.26 (t, J = 6.5 Hz, 1H), 2.13 (d, J = 5.8 Hz, 1H), 1.70–1.66 (m, 2H), 1.59–1.46 (m, 9H), 1.24 (brs, 20H), 0.87 (d, J = 7.2 Hz, 3H), and 0.82 (d, J = 6.5 Hz, 9H); ^13^C NMR (100 MHz, DMSO-d6) (Figures S11 and S12): δ 172.2, 172.1, 171.8, 171.7, 171.4, 68.5, 63.5, 60.7, 56.9, 51.1, 49.3, 40.3, 35.9, 35.8, 35.0, 33.5, 32.5, 29.0, 28.9, 28.8, 28.6, 28.5, 28.4, 28.1, 27.4, 25.4, 25.3, 24.9, 24.4, 24.0, 23.0, 21.4, 15.3, 10.7.

Compound **5** (12-hydroxyl octadecanoic acid-ILD-NH_2_). The following NMR data verified its structure. ^1^H NMR (DMSO-d6, 600 MHz) (Figure S13): 8.03–7.98 (m, 3H), 7.28 (brs, 1H), 7.05 (brs, 1H), 5.05 (brs, 1H), 5.30 (d, 1H), 4.39–4.35 (m, 1H), 4.24–4.21 (m, 1H), 4.13–4.11 (m, 1H), 4.14–4.11 (m, 1H), 2.16–2.09 (m, 2H), 1.74–1.70 (m, 1H), 1.63–1.57 (m, 2H), 1.49–1.42 (m, 5H), 1.22 (s, 22H), 0.86–0.78 (m, 12H); ^13^C NMR (DMSO-d6, 150 MHz) (Figures S14 and S15):; 172.7, 172.6, 172.4, 171.4, 80.3, 69.5, 57.1, 51.2, 49.6, 40.1, 37.1, 35.9, 35.1, 32.8, 31.3, 31.0, 29.1, 29.0, 28.9, 28.8, 28.5, 28.2, 25.3, 25.2, 24.4, 24.3, 24.0, 23.0, 22.0, 21.4, 15.4, 13.9, 13.8, 10.8. 

Compound **6** (hdaa-ILK-NH_2_). Its structure was verified by NMR data as follows. ^1^H NMR (600 MHz, DMSO-d_6_) (Figure S16): 7.97 (d, J = 7.5 Hz, 1H), 7.87 (d, J = 9.5 Hz, 6H), 7.75 (brs, 4H), 7.26 (brs, 1H), 7.03 (brs, 1H), 4.37 (t, J = 11.8 Hz, 1H), 4.29–4.25 (m, 1H), 4.16–4.11 (m, 2H), 3.98 (t, J = 5.5 Hz, 1H), 3.98 (t, J = 5.5 Hz, 1H), 3.36 (t, J = 6.5 Hz, 1H), 2.76–2.74 (m, 2H), 2.27–2.24 (m, 1H), 2.17–2.08 (m, 2H), 1.68–1.66 (m, 4H), 1.53–1.45 (m, 12H), 1.23 (s, 18H), 0.87 (d, J = 6.8 Hz, 2H), 0.81 (t, J = 11.8 Hz, 3H); ^13^C NMR (150 MHz, DMSO-d_6_) (Figures S17 and S18): 173.2, 172.3, 171.5, 171.3, 68.5, 63.5, 60.7, 56.9, 52.0, 51.1, 40.3, 38.6, 36.0, 35.1, 33.5, 32.5, 31.4, 29.0, 28.9, 28.7, 27.4, 26.6, 25.5, 25.4, 24.9, 24.4, 24.0, 23.0, 22.1, 21.4, 15.3, 10.7.

### 2.3. Collision Induced Dissociation MS/MS Spectral Fragmentation Ions Verified Structures of the Molecules Synthesized

The LC–MS/MS analysis of these amphiphiles validated their molecular masses and structures (Table 1). This was performed by C18 LC–MS/MS, as previously for caprylic-tripeptide conjugates [78,79].

**Figure 1:**
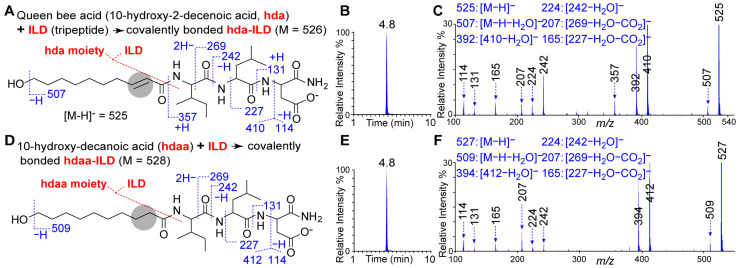
The molecular structures of typical hfa-aa1–aa2–aa3 amphiphiles, hda-ILD and hdaa-ILD, made via SPPS organic synthesis were confirmed by LC–MS/MS analysis. (**A**) Queen bee acid (hda) was covalently bonded to tri-peptide isoleucine (I)-leucine (L)-aspartic acid (D) (ILD), forming hda-ILD amphiphilic molecule. (**B**) LC–MS/MS chromatogram of hda-ILD. (**C**) LC–MS/MS spectrum of hda-ILD. (**D**) The hdaa bonded to ILD forming hdaa-ILD amphiphilic molecule. (**E**) LC–MS/MS chromatogram of hdaa-ILD. (**F**) LC–MS/MS spectrum of hdaa. Sciex Qtrap 6500^+^ MS/MS was used.

Compound **1** (hdaa-ILD-NH_2_). The structure of hdaa-ILD (M = 528 Daltons) was identified by the LC–MS/MS diagnostic fragmentation ions *m*/*z* 527 {the ion of deprotonated molecule: [M − H^+^] = (528 − 1 = 527)}, 509 [527 − H_2_O], 412, 394 [412 − H_2_O], 242, 224 [242 − H_2_O], 207, 165, 131, and 114 (Figure 1D–F, Table 1).

Compound **2** (3-hydroxyl decanoic acid-ILD-NH_2_). Its LC–MS/MS fragmentation ions verified the molecular mass M 528 Daltons and structure (*m*/*z* 527 [M − H^+^], 509, 399, 284, and 266) (Table 1).

Compound **3** (hda-ILD-NH_2_). Its LC–MS/MS fragmentation ions verified the molecular mass M 526 Daltons and structure (*m*/*z* 525 [M − H^+^]. 507 [525 − H_2_O]^−^, 410, 392 [410 − H_2_O]^−^, 357, 242, 224, 207, 165, 131, 114) (Figure 1A–C, Table 1).

Compound **4** (12-hydroxyl dodecanoic acid-ILD-NH_2_). Its LC–MS/MS fragmentation ions verified the molecular mass M 556 Daltons and structure (*m*/*z* 555 [M − H^+^], 537, 438, 423, 327, 282) (Table 1).

Compound **5** (12-hydroxyl octadecanoic acid-ILD-NH_2_). Its LC–MS/MS fragmentation ions verified the molecular mass M 640 Daltons and structure (*m*/*z* 639 [M − H^+^], 621, 524, 506, 394, 298) (Table 1).

Compound **6** (hdaa-ILK-NH_2_). Its LC–MS/MS fragmentation ions verified the molecular mass M 541 Daltons and structure (*m*/*z* 542 [M + H^+^], 524, 372, 171, 284, 259, 397, 146) (Table 1).

### 2.4. Prohealing Relevant Hydrogelability of Amphiphiles Covalently Modified from hda, hdaa, or Other hfa with Specific Tripeptides

The hydrogelation of these representative novel amphiphiles (1–6, Table 1) was undertaken in wound compatible phosphate-buffered saline (PBS) at 1.5% *w*/*v* concentration (0.75 mg in 0.5 mL PBS). We confirmed every hydrogel formation by the widely used vial inversion test [78,79]. These compounds were visually insoluble in PBS but attained solubility by adjusting the pH, then readjusting the pH back to 7.4. White precipitates (suspension) were then formed. The suspension transformed to hydrogel at a certain incubation time, i.e., gelation time (GT), after one minute sonication. All the compounds except compound **5** formed hydrogels within 30 min GT (Table 1). At 1.5% *w*/*v* and pH 7–7.5, the GT is shorter (*p* < 0.001) for compound 1 (12.3 min), with hydroxyl at C10 of the decanoyl, than compound **2** (30.4 min), with hydroxyl at C3 of the decanoyl moiety, suggesting that C10-hydroxyl is more favorable than C3-hydroxyl for hydrogelation (Table 1). The double-bond of 10-hydroxyl dec-2-enoyl moiety of compound **3** corresponded to a shorter GT (5.1 min) (*p* < 0.05) compared to compound **1** (12.3 min), where this double-bond did not exist (Table 1), which reflects the effect of C2 double-bond on hydrogelation. The π-electrons of the C2 double bond conjugate with the π-electrons of the C1 carbonyl double bond, delocalizing the electron density across the conjugated system, making the electron donor O atom of carbonyl in a hydrogen bond more electron-rich and thus increasing the strength of the hydrogen bond interaction; essentially, the conjugated system can stabilize the partial positive charge on the hydrogen bond donor, consequently contributing to hydrogelation. The GT is shorter for compound **1** (12.3 min) (*p* < 0.05), with a 10-hydroxyl decanoyl moiety, than for compound **4** (20.2 min) with a 12-hydroxyl-dodecanoyl moiety, suggesting that 10-hydroxyl decanoyl is better for hydrogelation than the 12-hydroxyl-dodecanoyl moiety when other molecular structural features are the same. The 12-hydroxyl dodecanoyl, with a longer hydrophobic carbon chain, should increase hydrophobicity and reduce aqueous solubility of the hfa-ILD-NH_2_ amphiphile more than the 10-hydroxyl-decanoyl. This could contribute to the longer GT of compound **4**. The hydrophobic domain with pertinent hydrophobicity is essential for an amphiphile to self-assemble into hydrogel. Too much or too little hydrophobicity can tip off the balance between the hydrophobicity and hydrophilicity of an amphiphile, resulting in failure in hydrogelation. This could explain why, in the same hfa-ILD-NH_2_ molecular model, compound **5** (12-hydroxyl octadecanoic acid-ILD-NH_2_) with 12-hydroxyl octadecanoyl moiety failed to hydrogelate, whereas compound **4** (12-hydroxyl dodecanoic acid-ILD-NH_2_) with a 12-hydroxyl-dodecanoyl moiety is gelable with a GT of 20.2 min (Table 1).

**Figure 2:**
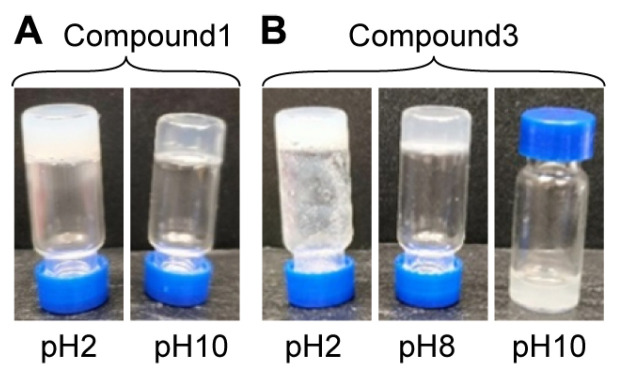
Hydrogels formed from hdaa-ILD-NH_2_ (**1**) and hda-ILD-NH_2_ (**3**) are stable in PBS at pH 2 to 10 and 2 to 8, respectively. (**A**) Photos showing that hdaa-ILD-NH_2_ (**1**) gelated and remained gelated in PBS at pH 2 (**left**) and 10 (**right**). (**B**) Photos showing that hda-ILD-NH_2_ (**3**) gelated and remained gelated in PBS at pH 2 (**left**) and 8 (**middle**). The hydrogel was generated from compound **1** or **3** at 1.5% *w*/*v* in PBS and transformed to solution phase at pH 11.5 or 10, respectively.

We further determined the gelability of compounds **1**–**4** at concentrations from 0.1% to 1% (*w*/*v*) using a vial inversion test. We observed that all four gelated at ≥0.5% *w*/*v* concentration in PBS but remained in solution at lower concentrations. Additionally, hydrogel formed at 0.5% *w*/*v* concentration were clear, while those formed at higher concentrations were opaque (Figure 2A, for compound **1**; Figure 2B, for compound **3**). This observation warrants a systematic study of the quantitative relationship between hydrogelation and concentration in the future.

We also examined the stability of these hydrogels at various pH levels, which encompass the pH range of human wounds under different treatments [90,91]. These hydrogels remained gelated from pH 2 to pH 8, demonstrating their ability to maintain stability within a patho-physiologically relevant pH range. The hydrogel of compound **1** at 1.5% *w*/*v* changed from opaque to more translucent when pH was increased from 2 to 10 (Figure 2A) and became a liquid solution at pH 11.5. In comparison, the hydrogel of compound **3** changed similarly when pH increased from 2 to 8 (Figure 2B) and dissolved at pH 10. The double-bond in 10-hydroxyl dece-2-noyl moiety of compound **3** reduced the tolerance from pH 10 to 8 for hydrogel stability, although it can reduce the gelation time (Table 1). The conjugation of the C2 double bond with C1 carbonyl double bond could be more sensitive to maximal pH, although it can enhance pro-geling hydrogen bonding.

### 2.5. Rheological and Shear-Thinning Properties of Hydrogels Self-Assembled from the Selected Novel Amphiphiles

We determined prohealing and antimicrobial related rheological and injectable shear-thinning properties of hydrogels that were self-assembled from selected novel amphiphiles covalently modified from hda and hdaa with ILD tripeptide. ILD was selected because we recently found that the caprylic acid-ILD-NH_2_ amphiphile is able to self-assemble to prohealing hydrogel that is wound-compatible, shear-thinning, injectable, non-toxic, and biodegradable [78,79]. Hydrogel injectability represents its ability to precisely fill 3D surfaces and cavities as a liquid and then solidify on-site as an elastic matrix, although this hydrogel can be applied to wounds by pipetting, coating, or spraying, other than by injection. The hydrogel is expected to comply with wound healing physiochemically and mechanically for the repair and regeneration of cells and tissue.

**Figure 3:**
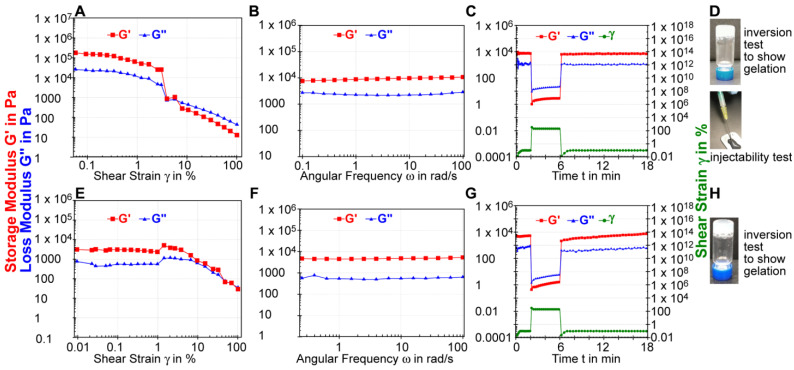
Gelation and rheological and injectable tests of hda-ILD-NH_2_ (**3**) and hdaa-ILD-NH_2_ (**1**) hydrogels. For hda-ILD-NH_2_ (**3**) hydrogels: (**A**) shear strain amplitude sweep test; (**B**) frequency sweep; (**C**) thixotropic test; and (**D**) photos: vial-inversion test for gelation (**upper**) and injectability test (**lower**). For hdaa-ILD-NH_2_ (**1**) hydrogels: (**E**) shear strain amplitude sweep test; (**F**) frequency sweep test; (**G**) thixotropic test; and (**H**) photo of vial-inversion test for gelation. Shear strain amplitude sweep test had a constant frequency of 10 rads/s. Frequency sweep test was with a constant shear strain of 1%. Thixotropic test was performed with imposition of a hydrogel-endurable shear strain of 0.1% and a hydrogel-breaking shear strain of 200%. Hydrogels were self-assembled from hda-ILD-NH_2_ (**3**) or hdaa-ILD-NH_2_ (**1**) at 3% (*w*/*v*) in PBS at pH 7.4 at 23 °C, and then were tested on an Anton-Paar MCR 092 model rheometer or injected from a syringe to surface at 37 °C.

The rheological properties of our novel hda-ILD-NH_2_ (**3**) hydrogel and hdaa-ILD-NH_2_ (**1**) hydrogel prepared from compounds **3** and **1** respectively (Table 1) were assessed using oscillatory rheology and are presented in Figure 3. The amplitude/shear strain sweep test for these hydrogels showed that storage modulus (G′) was higher than loss modulus (G″) in the limit of the linear viscoelastic region (LVE) (Figure 3A,E), indicating the presence of an elastic hydrogel structure. When the shear strain increased from 0.1% to 100%, the storage modulus (G′) and loss modulus (G″) slowly decreased until about 3% and 9% strain, respectively, for hda-ILD-NH_2_ (3) hydrogel and hdaa-ILD-NH_2_ (1) hydrogel, followed by a sharp decrease, and reached a point where G′ and G″ curves crossed over each other (crossover point G′ = G″). Further increase in strain leads to shear thinning where G′ < G″ for hda-ILD-NH_2_ (**3**) hydrogel, indicating a transition from the hydrogel state to a viscous liquid state, whereas this transition did not clearly appear for hdaa-ILD-NH_2_ (**1**) hydrogel. The hda-ILD-NH_2_ (**3**) hydrogel showed the highest value (G′ = 100,000 Pa) for storage modulus (G′) and loss modulus (G″). The frequency sweep experiment (Figure 3B,F) was carried out at a constant 1% strain (within the LVE range) with a frequency range from 100–0.1 rad/s; both hydrogels showed a hydrogel status with constant values for storage modulus and loss modulus, where G′ is more than G″ and independent of the frequency, reflecting the high stability of these hydrogels; both hydrogels had storage G′ moduli between ~1 k and ~10 k Pa, compatible to skin tissue [92,93]. The elastic non-Newtonian nature [94] of these hydrogels further supports their suitability for wound treatment hydrogel and gel formulations, where the ideal rheological viscoelastic moduli are typically within a range where the storage modulus (G′) is between 1–100 kPa to mimic the elastic properties of healthy skin [95]. We prefer a narrower range of G′, ~0.2–17 kPa, as the elastic moduli of the wound exocellular matrix and health dermis and sub-dermis are between that of brain (0.2–1 kPa) and muscle (8–17 kPa) [92,93,96].

The shear-thinning properties of hda-ILD-NH_2_ (**3**) hydrogel and hdaa-ILD-NH_2_ (**1**) hydrogel were examined using time-dependent shear thinning thixotropic tests (Figure 3C,G). In this experiment, the shear strain was suddenly increased from 0.1% to 200% at 2 min, stayed at 200% for 4 min, and then was reduced back to 0.1%. The G′ and G″ values remained constant when 0.1% strain was applied for the initial 2 min, then the G′ and G″ values dropped abruptly when 200% strain was imposed. It is worth noting that G″ was more than G′, where the fluid turned into solution and remained as a solution until the 200% strain was removed. These changes revealed the rapid shear-thinning properties of both hydrogels, as the hydrogels were liquefied by the increased shear strain. The G′ and G″ values completely switched back to their original values when the 0.1% strain was restored. The shear-thinning and regenerative properties of both hydrogels provided mechanistic insights into their injectability. To directly determine the hydrogel injectability, we liquefied each hydrogel by shaking, drew the liquid into a syringe through a 25-gauge needle, and then injected the liquid into the target site. We observed spontaneous re-gelation, as demonstrated in the photo of this process (Figure 3D lower panel). Taken together, our studies unveiled that hda-ILD-NH_2_ (**3**) hydrogel and hdaa-ILD-NH_2_ (**1**) hydrogel are both shear-thinning, non-Newtonian [94], and injectable; further confirming their suitability to accelerate wound healing and inhibit infection, these hydrogels can freely flow as liquid under attainable shear-strain, enabling them to penetrate wound cavities to access and act on bacteria more thoroughly than conventional antimicrobials.

### 2.6. Determination of Gelation-Related Fibrous Structures Using Field Emission Scanning or Transmission Electronic Microscopy

**Figure 4:**
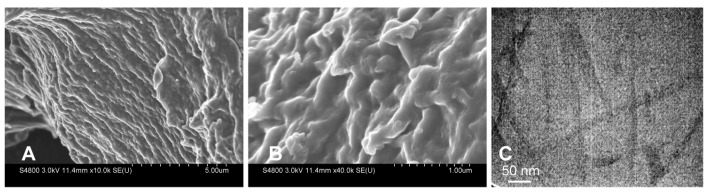
Fibrous structures of hda-ILD-NH_2_ (**3**) determined using field emission scanning and transmission electron microscopy (FE–SEM and TEM). (**A**) Image at ×10,000 and (**B**) image at ×40,000 for FE–SEM. (**C**) The TEM image at ×60,000 magnification. FE–SEM secondary electrons were detected using an upper detector, with a working distance of 11.4 mm and acceleration voltage of −3.0 kV.

To determine the hydrogelation-related fibrous structures formed from hfa-ILD-NH_2_, we conducted a field emission scanning and transmission electronic microscopy (FE–SEM and TEM) study of the representative structure, i.e., hda–ILD–NH_2_ (**3**). FE–SEM imaging at ×10,000 magnification (Figure 4A) revealed the upper layer of fibrous networks in the hydrogel. At ×40,000 magnification, the SEM image showed that the networks consisted of fibrils longer than 1 µm, similar to those reported by other researchers (Figure 4B) [97,98]. The TEM study revealed the nano-fibrous rod-like structures formed from hda-ILD-NH_2_ (**3**) at ×60,000 magnification (Figure 4). There is a high propensity for hda-ILD-NH_2_ (**3**) molecules to form fibrils through intermolecular hydrogen bonds between N-H (hydrogen bonding donor) and C=O (hydrogen bonding acceptor) groups, as it contains four N-H groups, one hydroxy group, and 5 C=O groups, forming hydrogen bonds along its backbone (Table 1). Additionally, the nonpolar side chains of I and L, along with the hydrocarbon tail of hda in hda-ILD-NH_2_ (**3**), can interact with the corresponding groups of another (**3**) through hydrophobic interactions, thereby promoting fibril formation [99].

### 2.7. The Evaluation of Antibacterial Activity

**Figure 5:**
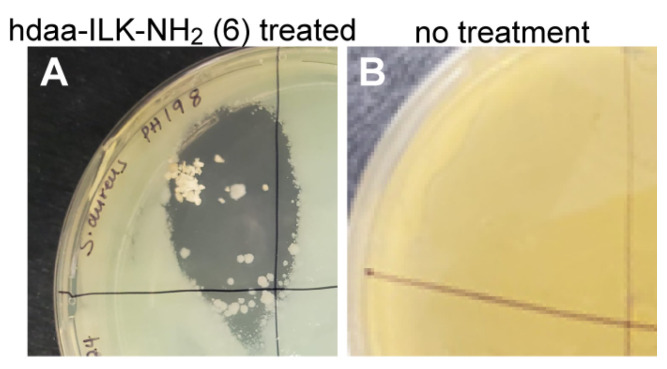
The amphiphile hdaa-ILK-NH_2_ (**6**) modified from hdaa with tripeptide ILK-NH_2_ by peptide bonding inhibited *S. aureus* in a Kirby–Bauer disc diffusion assay. (**A**) *S. aureus* was inoculated and 5 mg hdaa-ILK-NH_2_ (**6**) was added to the well. (**B**) *S. aureus* without treatment. The inhibition zone of *S. aureus* was photographed and measured at 25 mm in mean radius.

We explored the antimicrobial activity of hdaa-ILK-NH_2_ (**6**) described in Table 1 against *S. aureus* using the standard Kirby–Bauer disc diffusion method. We found that hdaa-ILK-NH_2_ (**6**) markedly inhibited *S. aureus* in the culture dish with 25 mm mean radius of the inhibition zone (Figure 5). This observation indicates that the amphiphile hdaa-ILK-NH_2_ (**6**) modified from hdaa with tripeptide ILK-NH_2_ by peptide bonding had a bacteriostatic action against strains of *S. aureus*. It is likely also to be antimicrobial against other bacteria, as well as bactericidal and/or antibiofilm, as it is a unique amphiphile that tends to interrupt the bacteria membrane, based on its structure. Additional amphiphiles that we designed and synthesized by the modification of hda, hdaa, and other hydroxyl fatty acids with specific tripeptides could also show antimicrobial activities. These warrant further investigation in the future. Bacteriostatic action of hdaa-ILK-NH_2_ (**6**) or other potential amphiphiles against strains of *S. aureus* is significant because it inhibits bacterial growth, allowing the immune system to effectively target and eliminate the bacteria. It has the potential to help manage antibiotic resistance by reducing selective pressure for resistant strains and can be used in combination with bactericidal agents to enhance treatment efficacy. The amphiphiles developed in this study are likely to be non-toxic, as they consist of non-toxic queen bee acid or another hydroxyl fatty acid covalently bonded to a tripeptide. Thus, they are likely to be safe to humans and animals.

## 3. Materials and Methods

### 3.1. Materials

Fmoc-protected amino acids, i.e., Fmoc-Ile-OH (CAS No. 71989-23-6, 98%), Fmoc-Leu-OH (CAS No. 35661-60-0, 99.78%), Fmoc-Asp(OtBu)-OH (CAS No. 71989-14-5, 99.92%), and fatty acids, i.e., 10-hydroxydecanoic acid (CAS No. 1679-53-4, 99.77%), 3-hydroxydecanoic acid (CAS No. 14292-26-3, 99.25%), (*E*)-10-hydroxydec-2-enoic acid (CAS No. 14113-05-4, 98.0%), 12-hydroxydodecanoic acid (CAS No. 505-95-3, 97%), were obtained from BLD Pharmatech Co., Ltd. (Cincinnati, OH, USA) and used without further purification. 12-Hydroxyl stearic (octadecanoic) acid (CAS No. 106-14-9, 97%), was provided by Chemsavers Inc. (Bluefield, VA, USA). Fmoc rink amide resin (0.57 mmol/g, 100–200 mesh), the Kaiser test kit (Catalog no. KGZ001), and O-benzotriazole-N, N, N’ and N’-tetramethyluronium-hexafluoro-phosphate (HBTU, CAS No. 94790-37-1) were obtained from Aapptec, LLC (Louisville, KY, USA). Hydroxy-benzotriazole (HOBt, CAS No. 2592-95-2, 98.75%) was purchased from Apexbio (Houston, TX, USA). Trifluoroacetic acid (TFA; CAS No. 76-05-1, 99%) was acquired from Honeywell Research Chemicals (Muskegon, MI, USA). Piperidine (CAS No. 110-89-4, 99%) was obtained from Sigma-Aldrich Co., LLC (St. Louis, MO, USA). N, N-diisopropylethylamine (DIPEA, CAS No. 7087-68-5, 99%) was obtained from TCI America (Portland, OR, USA). N, N-dimethyl-formamide (DMF; CAS No. 68-12-2), diethyl ether (DE; CAS No. 60-29-7), and dichloromethane (DCM; CAS No. 75-09-2) were purchased from Thermo-Scientific (Ward Hill, MA, USA).

### 3.2. Synthesis and Structural Analysis of an Amphiphile Modified from a Hydroxyl Fatty Acid with a Tripeptide via Peptide Bonding

The amphiphile modified from a hydroxyl fatty acid with a tripeptide was synthesized using Fmoc-based solid-phase peptide synthesis (SPPS) strategies manually using PolyPrep columns obtained from Bio-Rad Laboratories (Hercules, CA, USA), similar to our synthesis of fatty acid-tripeptide conjugates [78,79]. Briefly, the synthesis was conducted on a 0.1 mmol scale using Fmoc-Rink amide resin. Fmoc-Rink amide resin (175 mg) was first swollen in DCM, 5.0 mL within a Biorad column for 30 min. The solvent was then drained, and 20% piperidine in DMF, 5.0 mL was added. The mixture was agitated for 20 min to remove the Fmoc protecting group. The resin was subsequently washed with DMF (3 × 5 mL) and DCM (3 × 5 mL). The successful deprotection of the Fmoc group was confirmed by a positive Kaiser test.

Next, Fmoc-Asp(tBu)-OH (164 mg) was dissolved in DMF (5.0 mL) along with HBTU (152 mg), HOBt (54 mg), and DIPEA (0.2 mL) in a scintillation vial. The mixture was sonicated for 1 min before being added to the resin column, which was shaken on a vortex mixer for 6 h. Following the coupling step, the solvent was removed, and the resin was washed with DMF (3 × 5 mL) and DCM (3 × 5 mL). The completion of the amino acid coupling was confirmed by a negative Kaiser test. To cap any unreacted sites, the resin was treated with a solution of acetic anhydride and pyridine (3:2, *v*/*v*; 5.0 mL) and rocked for 1 h. The resin was then washed thoroughly with DMF (3 × 5 mL) and DCM (3 × 5 mL). The Fmoc group was removed by treating the resin with 20% piperidine in DMF (5.0 mL) for 20 min, followed by washing with DMF (3 × 5 mL) and DCM (3 × 5 mL). Subsequent coupling of the second and third amino acids, as well as the hda, hdaa, or other hfa, followed the same protocol of Fmoc deprotection, activation, and coupling. Cleavage of the fatty acid–peptide conjugate from the resin was performed by treating it with a mixture of trifluoroacetic acid (TFA), water, and tri-isopropyl-silane (TIPS) (5.0 mL; 95:2.5:2.5, *v*/*v*) at room temperature for 2 h. The resin was filtered, and the filtrate was evaporated using a rotary evaporator to remove excess TFA. The crude product was precipitated by adding ice-cold diethyl ether, yielding a white solid. This precipitate was filtered through a sintered funnel and washed three times with cold diethyl ether. To replace the trifluoroacetate counter-ion with hydrochloride, the precipitate was dissolved in 0.1 M HCl solution (5.0 mL) and stirred for 15 min. Acetonitrile (5.0 mL) was then added, and the solution was dried in a dry ice bath before being lyophilized overnight using a freeze-dryer (Thermo Savant, Holbrook, NY, USA), producing a white powder. The structure of the amphiphile, a hydroxyl fatty acid linked to a tripeptide via peptide bonds, was confirmed using LC–MS/MS analysis.

### 3.3. Instrumental Molecular Analysis

#### 3.3.1. LC–MS/MS Method

The reagents and each amphiphile molecule modified from a hydroxyl fatty acid with a tripeptide were analyzed using an LC–MS/MS system consisting of an Agilent 1100 LC system (HPLC-DAD-autosampler, Agilent Technologies, Inc., Santa Clara, CA, USA) and a QTRAP 6500^+^ quadruple-linear trap mass spectrophotometer with electrospray ionization (Sciex.com, Framingham, MA, USA).

#### 3.3.2. NMR Method

The NMR analysis was carried out using a Brucker 600 or 400 MHz NMR instrument, DMSO-d_6_ (CAS No. 2206-27-1, 99.9 atom% D, Thermo-scientific, Fair Lawn, NJ, USA) as a solvent, and Topspin 4.3.0 version software. The ^1^H NMR (400 or 600 MHz), 13C NMR (100 or 150 MHz), and 13C DEPT data were acquired using a purified compound (20 mg) dissolved in 0.7 mL DMSO-d_6_ in a 5 mm diameter NMR tube. DEPT-135 determined the multiplicity of carbon atoms; CH_2_ groups had inverted signals, whereas the CH and CH_3_ groups were upright, and the quaternary carbon (C) did not have any signal.

### 3.4. Hydrogelation Tests

#### 3.4.1. Hydrogel Preparation and Test

Each lyophilized amphiphile molecule modified from a hydroxyl fatty acid with a tripeptide was dissolved in PBS at a final concentration of 0.5% (5.0 mg in 1 mL PBS), 1.5% (15.0 mg in 1 mL PBS), and 3% (30.0 mg in 1 mL PBS. To dissolve the compounds, the pH of the peptide solutions was raised to 9.0 by adding 0.1 M NaOH, then adjusted back to pH 7.4 by slowly adding 0.1 N HCl, followed by sonication. After sonication, the amphiphile quickly formed a suspension, which was left at room temperature to allow hydrogelation. Hydrogelation was confirmed using the vial inversion test and recorded with photographs.

#### 3.4.2. Sterile Conditions

PBS, 0.1 N HCl, 0.1 M NaOH, pipette tips, and Eppendorf tubes were autoclaved at 130 °C for 45 min using a Steris AMSCO 250 LS autoclave. All other steps for hydrogel formation were performed under pathogen-free conditions inside a BSL-2 hood.

### 3.5. Rheological Test Procedures

The experiments were performed on 50 µL hydrogel samples using an Anton Paar MCR 092 rheometer (Anton Paar USA, Inc., Houston, TX, USA), equipped with a 20 mm cone plate and a 39 µm gap. To investigate the effect of concentration on hydrogel strength and viscoelastic properties, amplitude/strain sweep tests were conducted using oscillatory shear strain. Storage (G′) and loss (G″) moduli were measured across a strain range of 0.01 to 100% at a constant frequency of 10 rad/s. To evaluate mechanical stability, frequency sweep tests were performed at angular frequencies from 1 to 100 rad/s, with a constant 1% strain within the linear viscoelastic region identified from the amplitude sweep. The hydrogel structure remained intact under these conditions. Thixotropic properties were studied to examine the hydrogels’ shear-thinning behavior over time. When subjected to a high shear strain of 200% for 4 min, the hydrogel liquefied, and re-gelation occurred once the shear strain was reduced to 0.1%. The G′ and G″ values indicated the hydrogels’ elastic, hydrogel-like behavior and their viscous, liquid behavior, respectively. [100].

### 3.6. Procedures for Field Emission Scanning or Transmission Electronic Microscopy

The 3% *w*/*v* hydrogel was freeze-dried at −80 °C and lyophilized under vacuum to produce a fine powder. FE–SEM analysis was performed on the lyophilized hydrogel using an S4800 field emission scanning electron microscope (Hitachi, Santa Clara, CA, USA) under high vacuum conditions to examine the surface structure of the freeze-dried peptide-based hydrogel, following established procedures [97,98]. The parameters used were stage distance of 12 mm, acceleration voltage of −3.0 kV, and working distance of 11.4 mm. The sample was sputter-coated with a thin carbon layer to enhance conductivity. High magnification revealed the fibrous network on the top layer of the hydrogel samples.

The procedures for TEM analysis are as follows. Hydrogels dissolved with water were drop-casted (1 μL) onto carbon-coated 300 mesh carbon grids (Ted Pella Inc., Redding, CA, USA) and then air-dried for 10 min. The carbon film was washed 3 times with deionized water drops and the grids were negatively stained with 2% uranyl acetate (VWR LLC, Philadelphia, PA, USA) in water and air-dried. Stained grids were examined under a JEOL 2010 transmission electron microscope (operated at 200 keV) and TEM images were acquired.

### 3.7. Antibacterial Activity Assessment by Kirby–Bauer Disc Diffusion Method

The antibacterial activity of hdaa-ILK-NH_2_ (**6**) against *S. aureus* was assayed by the agar disc diffusion method, the Kirby–Bauer disc diffusion method, following the published procedures [101]. Nutrient Agar (NA) medium was used to cultivate *S. aureus* (stain: Zen 29). *S. aureus* suspension was diluted and adjusted to the equivalent of the 0.5 McFarland standard (~1 × 10^8^ CFU/mL) and then inoculated to the plates (100 µL/plate). A hydrogel puncture was used to generate 6 mm diameter wells. The hdaa-ILK-NH_2_ (**6**) (5 mg) was added to the well. The plates with and without treatment were incubated at 37 °C for 24 h. The inhibition of *S. aureus* was assessed visually based on the size of the clear zone surrounding the well and recorded as the mean radius in millimeters.

### 3.8. Statistical Analysis

Statistical analysis was performed using GraphPad Prism 9.0 (GraphPad, Boston, MA, USA). The data were evaluated through repeated measures analysis of variance. Pairwise comparisons among peptide groups were adjusted using the Tukey method. A *p*-value of ≤0.05 was considered statistically significant. Results are presented as mean ± standard deviation.

## 4. Conclusions

We have developed novel amphiphiles by modifying the major reparative and antimicrobial components hda and hdaa of MGH/RJ with specific tripeptides via peptide bonding through a rational design and organic synthesis. Our molecular design incorporated amphiphile targets which were biocompatible in wound healing, biodegradable, non-toxic, hydrogelable in wound niches, prohealing, and antimicrobial. The molecules were designed in an hda(hdaa)-aa1-aa2-aa3 structural model with rational selection criteria for each moiety in its short sequence. We synthesized these amphiphiles using Fmoc/tBu-based SPPS and verified their structures by NMR and LC/MS/MS. Among these amphiphiles, we tested several containing hda, hdaa, ILD-NH_2_, ILK-NH_2_, and we found them suitable for promoting wound healing and inhibiting infections. They are capable of forming hydrogels with stable elastic moduli and injectable shear-thinning properties pertinent for cell and tissue repair and regeneration, and for infection control. The fibrous nanostructures self-assembled from hda-ILD-NH_2_ (**3**) were examined using TEM, which are likely to be the initial fibrils, which further interact with each other to form the hydrogel network. This will be further investigated by circular dichroism in the future. The self-assembling hydrogelation of other amphiphiles synthesized in this study will also need to be depicted using TEM and circular dichroism. Our assay demonstrated that hdaa-ILK-NH_2_ (**6**) markedly inhibited *S. aureus*. Comprehensive studies are needed in the next phase of our approach to investigating the pro-healing and antimicrobial properties of both the tested and untested amphiphiles synthesized in this report.

## 5. Patents

Hong, S.; Baravkar, S.B.; Lu, Y. Amphiphilic Conjugates of Fatty Acids or their Derivatives. US Provisional Patent No: 63617303 2024.

## Figures and Tables

**Table 1:** Representative novel amphiphiles generated by hydroxy fatty acid–tripeptide conjugates reported in this work: chemical structures, MS/MS ions, hydrogelation features, and gelation times.

Comp#	Structure and Sequence ^(a)^	MS/MS Ions, *m*/*z* ^(b)^	Able to Hydro Gelate? ^(c)^	Gelation Time, Min ^(d)^
**1**	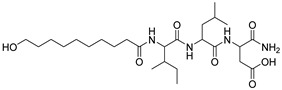 hdaa-ILD-NH_2_	527 [M − H^+^], 509, 412, 394, 242, 224, 207, 165, 131, 114	yes	12.3 ± 1.8
				*p* < 0.001 vs. #2;
				*p* < 0.05 vs. #3;
				*p* < 0.05 vs. #4;
				*p* < 0.001 vs. #6
**2**	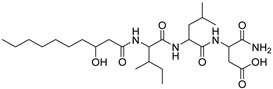 3-hydroxyl decanoic acid-ILD-NH_2_	527 [M − H^+^], 509, 399, 284, 266	yes	30.4 ± 3.8
				*p* < 0.001 vs. #3
				*p* < 0.01 vs. #4
**3**	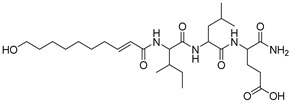 hda-ILD-NH_2_	525 [M − H^+^]. 507, 410, 392, 357, 242, 224, 207, 165, 131, 114	yes	5.1 ± 1.0
				*p* < 0.001 vs. #4;
				*p* < 0.001 vs. #6
**4**	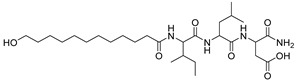 12-hydroxyl dodecanoic acid-ILD-NH_2_	555 [M − H^+^], 537, 438, 423, 327, 282	yes	20.2 ± 2.9
				*p* < 0.01 vs. #6
**5**	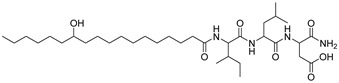 12-hydroxyl octadecanoic acid-ILD-NH_2_	639 [M − H^+^], 621, 524, 506, 394, 298	Not	Infinite
**6**	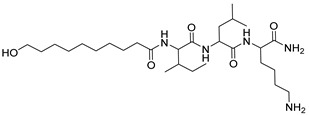 hdaa-ILK-NH_2_	542 [M + H^+^], 524, 372, 171, 284, 259, 397, 146	yes	30.4 ± 2.6

^(a)^ C-terminus, amidated; N-terminus, hydroxyl fatty acid acylated; hda, queen bee acid (10-hydroxy-2-decenoic acid); hdaa, 10-hydroxydecanoic acid; letters I, L, D, and K denote amino acids: I—isoleucine, L—leucine, D—aspartic acid, K—lysine; NH_2_- C-terminus amide. ^(b)^ MS/MS ion acquired by LC–MS/MS, single charged (z = 1, *m*/*z* = Daltons); ^(c)^ Compound concentration, 1.5% *w*/*v*; pH in PBS saline, 7.0–7.5; ^(d)^ The gelation time, expressed in minutes, is presented as the mean ± SD, with *n* = 3 independent tests. “#” stands for “number”.

## Data Availability

Data are contained within the article.

## Supplementary Materials

The following supporting information can be downloaded at: https://www.mdpi.com/article/10.3390/molecules30030615/s1, Table S1: Additional novel amphiphiles synthesized in this study. Figures S1–S27. 1H, 13C, and 13C DEPT 135 NMR spectra.
