# Supplementary material for: Molecular Modification of Queen Bee Acid and 10-Hydroxydecanoic Acid with Specific Tripeptides: Rational Design, Organic Synthesis, and Assessment for Prohealing and Antimicrobial Hydrogel Properties
Source: Molecules. 2025 Jan 30;30(3):615. doi: 10.3390/molecules30030615 (PMC11819776; doi:10.3390/molecules30030615)
Supplement: Supplementary file 1 [file molecules-30-00615-s001.zip › molecules-3440632-supplementary.pdf]

Supplementary Materials

Molecular Modification of Queen Bee Acid and 10-Hydroxydecanoic Acid with Specific Tripeptides: Rational Design, Organic Synthesis, and Assessment for Prohealing and Antimicrobial Hydrogel Properties

Song Hong[1,2,*], Sachin B. Baravkar[1], Yan Lu[1], Abdul-Razak Masoud[1], Qi Zhao[3], Weilie Zhou[4]

[1] Neuroscience Center of Excellence, School of Medicine, Louisiana State University Health, New Orleans, LA 70112, U.S.A.
[2] Department of Ophthalmology, School of Medicine, Louisiana State University Health, New Orleans, LA 70112, U.S.A.
[3] NMR Laboratory, Department of Chemistry, Tulane University, New Orleans, LA 70115, U.S.A.
[4] Department of Physics & AMRI, University of New Orleans, LA 70148, U.S.A.
* Correspondence: Song Hong, PhD, email: shong@lsuhsc.edu

Table S1. Additional novel amphiphiles synthesized in this study.

**comp#**	**structure and sequence** [(a)]	**MS/MS ions, m/z**[(b)]
7	hda-ILK-NH_2	540 [M + H^+], 522, 395, 372, 282, 259, 169, 146
8	hdaa-ILR-NH_2	570 [M + H^+], 552, 400, 397, 287, 284, 174, 171
9	hda-ILR-NH_2	568 [M + H^+], 550, 400, 395, 287, 282, 174, 169
10	hdaa-IQK-NH_2	557 [M + H^+], 387, 412, 284, 274, 171, 146
11	hda-IQK-NH_2	555 [M + H^+], 537, 387, 410, 282, 274, 169, 146

Notes: (a) C-terminus, amidated; N-terminus, hydroxyl fatty acid acylated; hda, queen bee acid (10-hydroxy-2-decenoic acid); hdaa, 10-hydroxydecanoic acid; letters I, L, K, Q, and R denote amino acids: I – isoleucine, L – leucine, K – lysine, Q – glutamine, R-arginine; NH_2- C-terminus amide. (b) MS/MS ion acquired by LC-MS/MS, single charged (z = 1, m/z = Daltons, M = molecular mass, Daltons).

Supplementary Figures. S1 to S27. ^{1}H, ^{13}C, and ^{13}C DEPT 135 NMR spectra

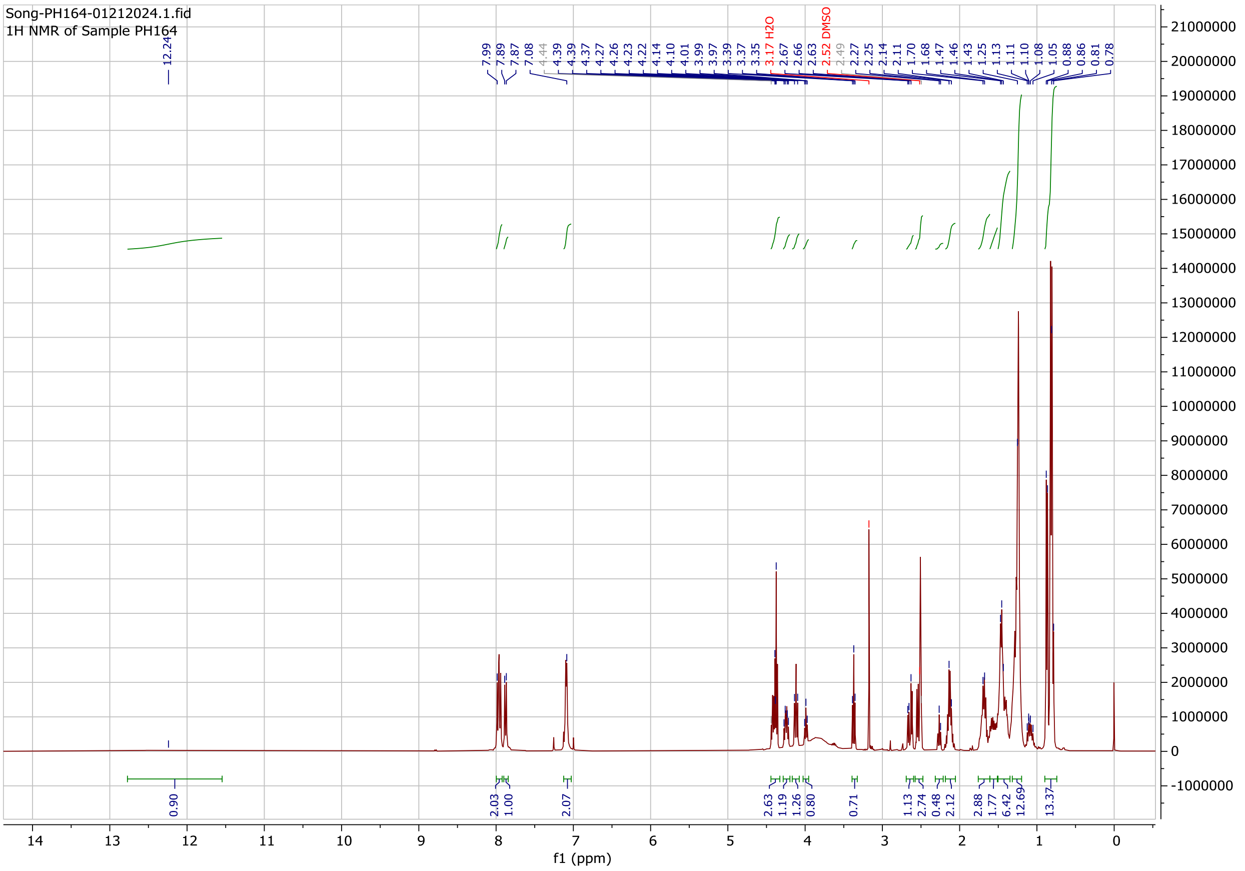

Figure S1. ^{1}H NMR spectrum of compound **1** (**hdaa-ILD-NH**$_2$, PH164) in DMSO-d6.

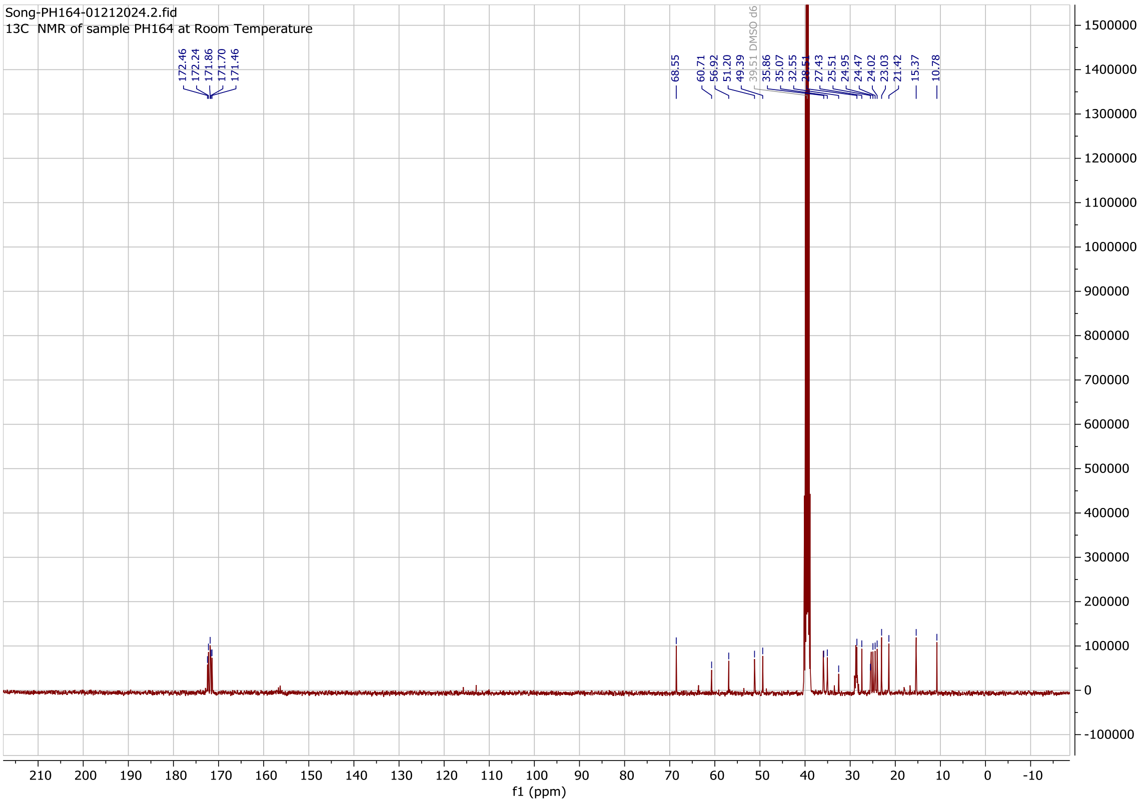

Figure S2. ^{13}C NMR spectrum of compound **1** (**hdaa-ILD-NH**$_2$, PH164) in DMSO-d6.

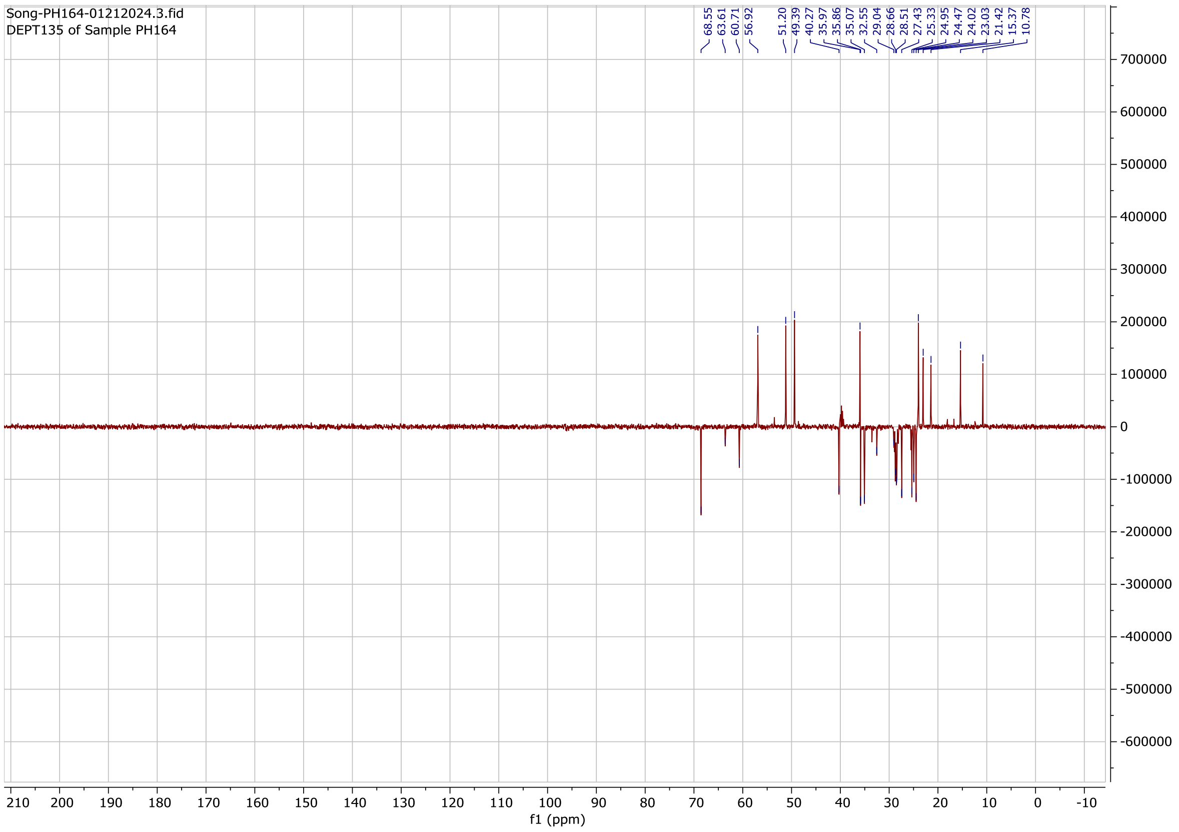

Figure S3. ^{13}C DEPT NMR spectrum of compound 1 (**hdaa-ILD-NH$_2$**, PH164) in DMSO-d6.

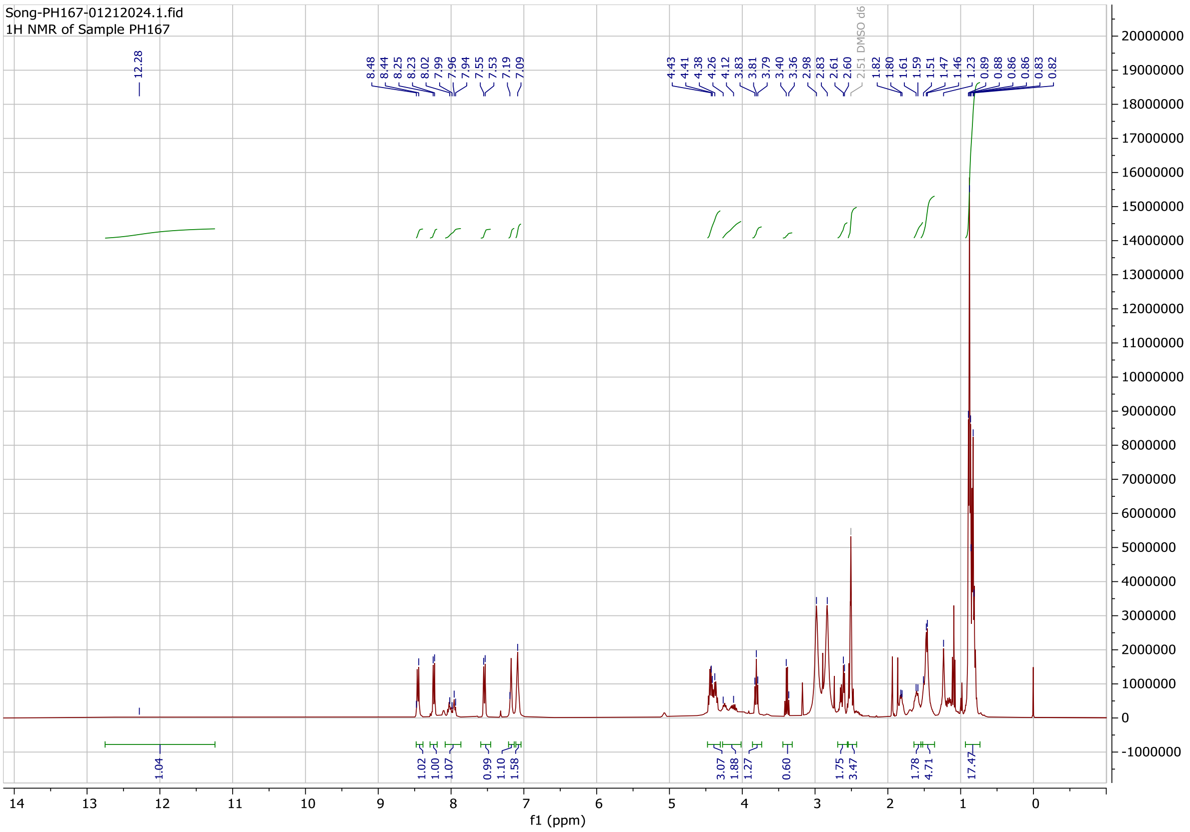

Figure S4. ^{1}H NMR spectrum of compound **2 (3-hydroxyl decanoic acid-ILD-NH$_2$**, PH167) in DMSO-d6.

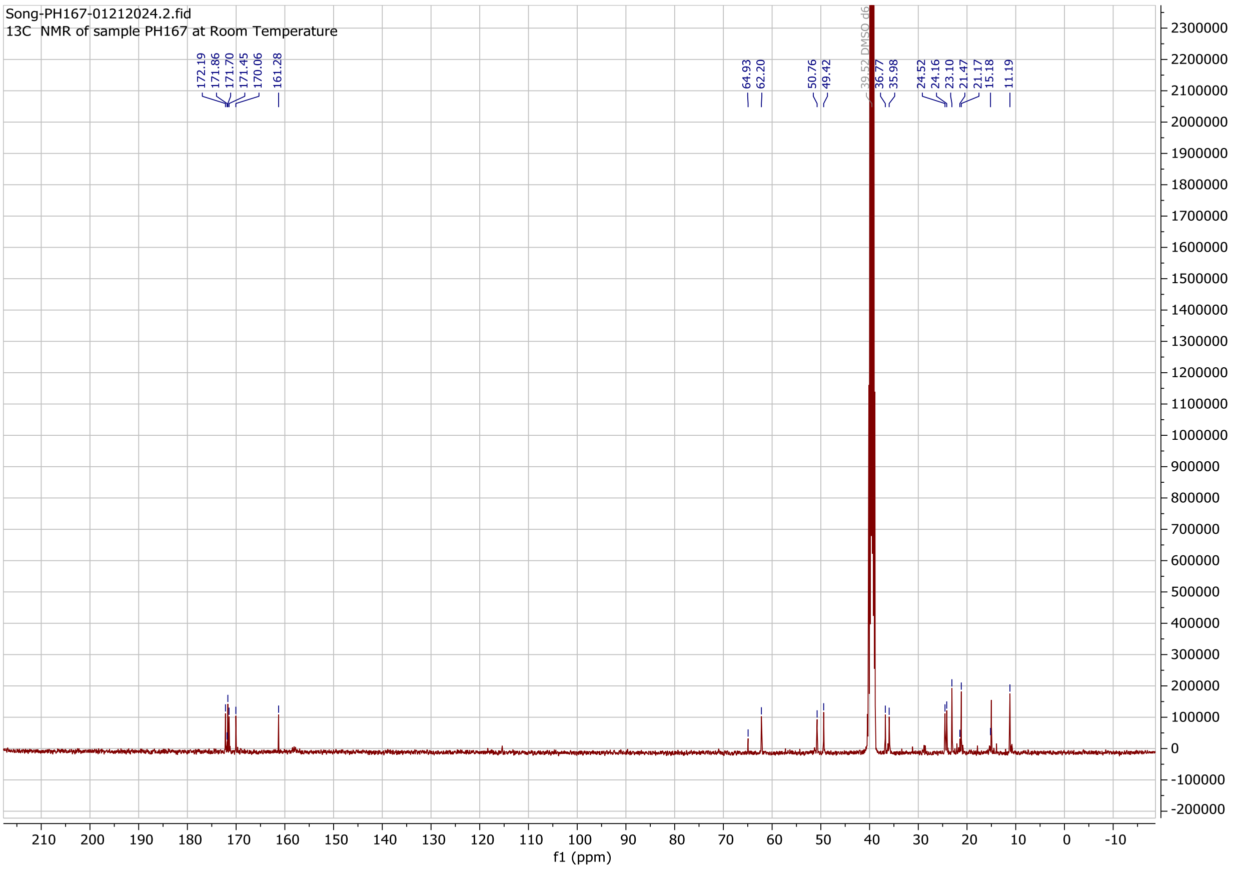

Figure S5. ^{13}C NMR spectrum of compound **2** (**3-hydroxyl decanoic acid-ILD-NH$_2$**, PH167) in DMSO-d6.

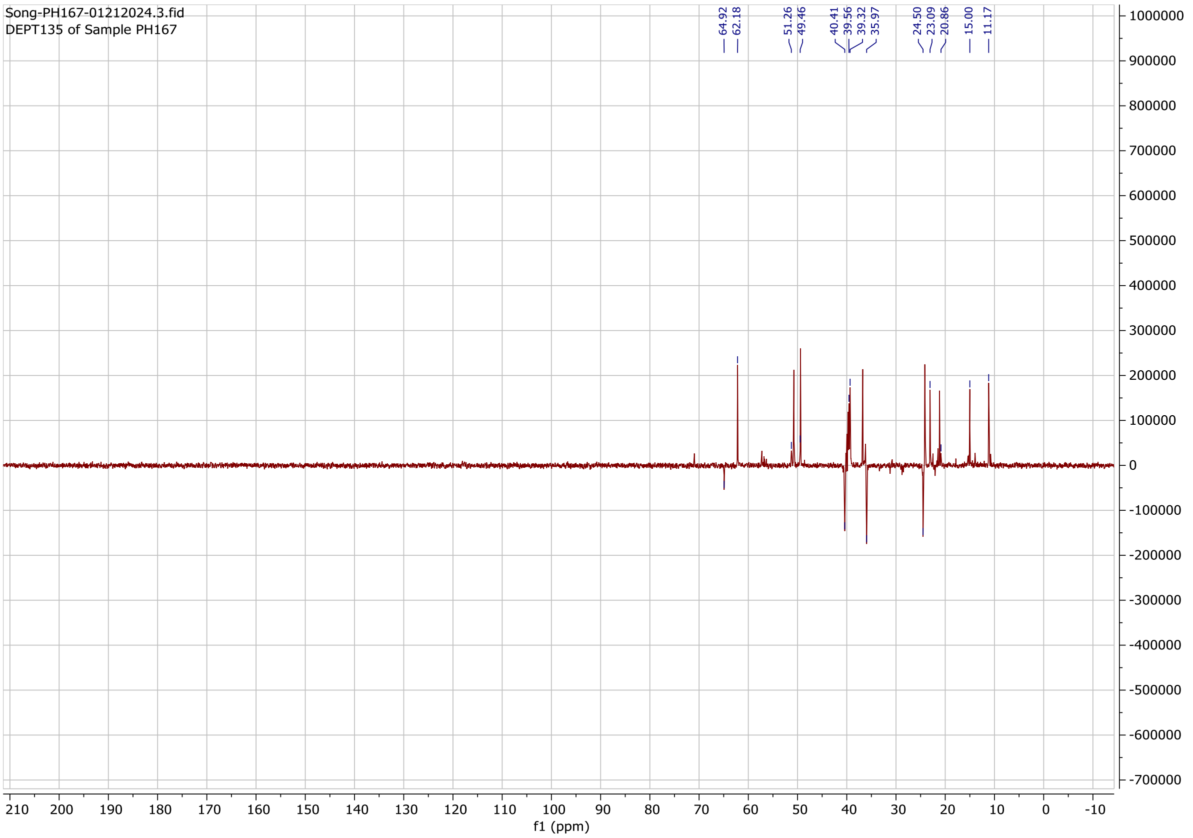

Figure S6. ^{13}C DEPT NMR spectrum of compound 2 (**3-hydroxyl decanoic acid-ILD-NH$_2$**, PH167) in DMSO-d6.

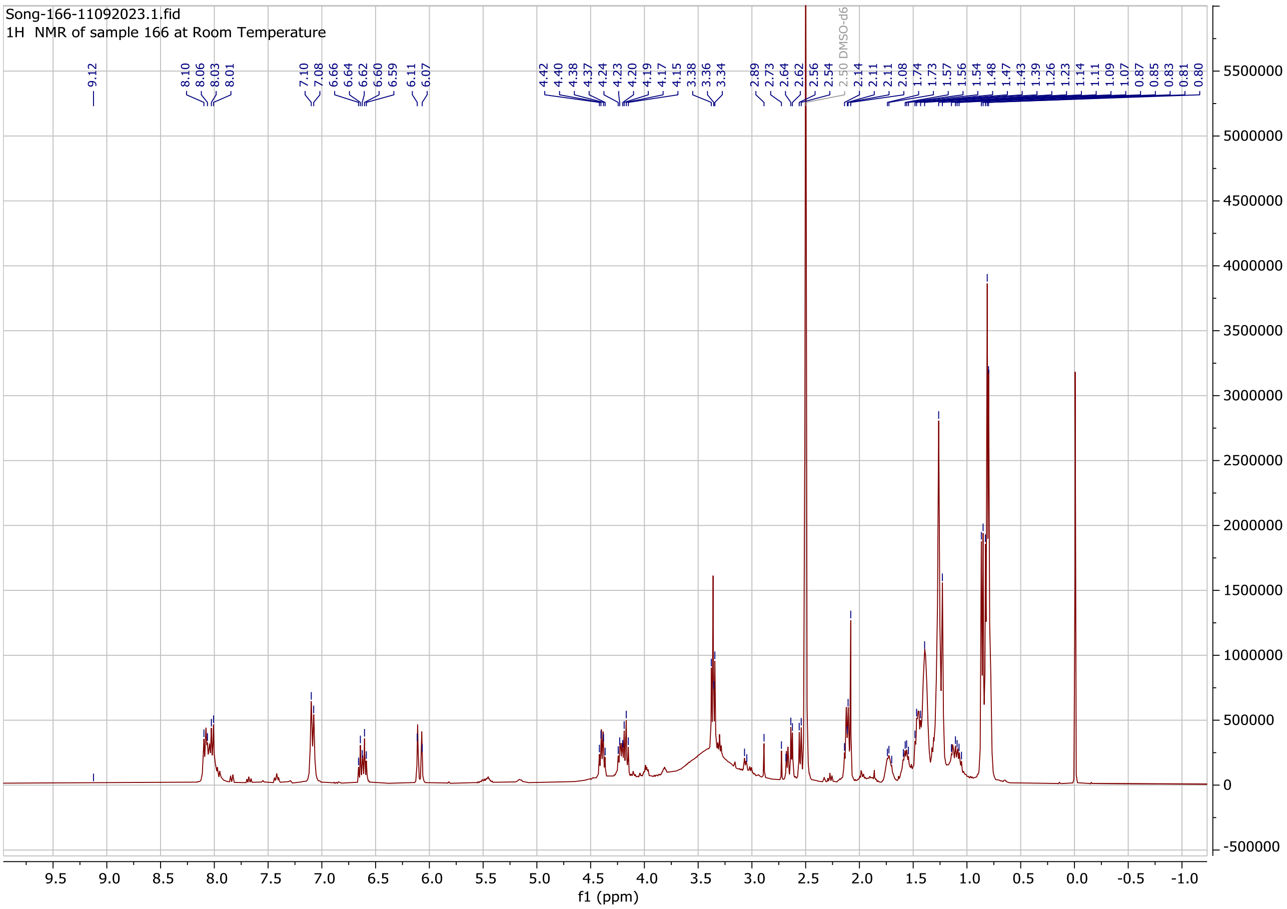

Figure S7. ^{1}H NMR spectrum of compound **3** (**hda-ILD-NH$_2$**, PH166) in DMSO-d6.

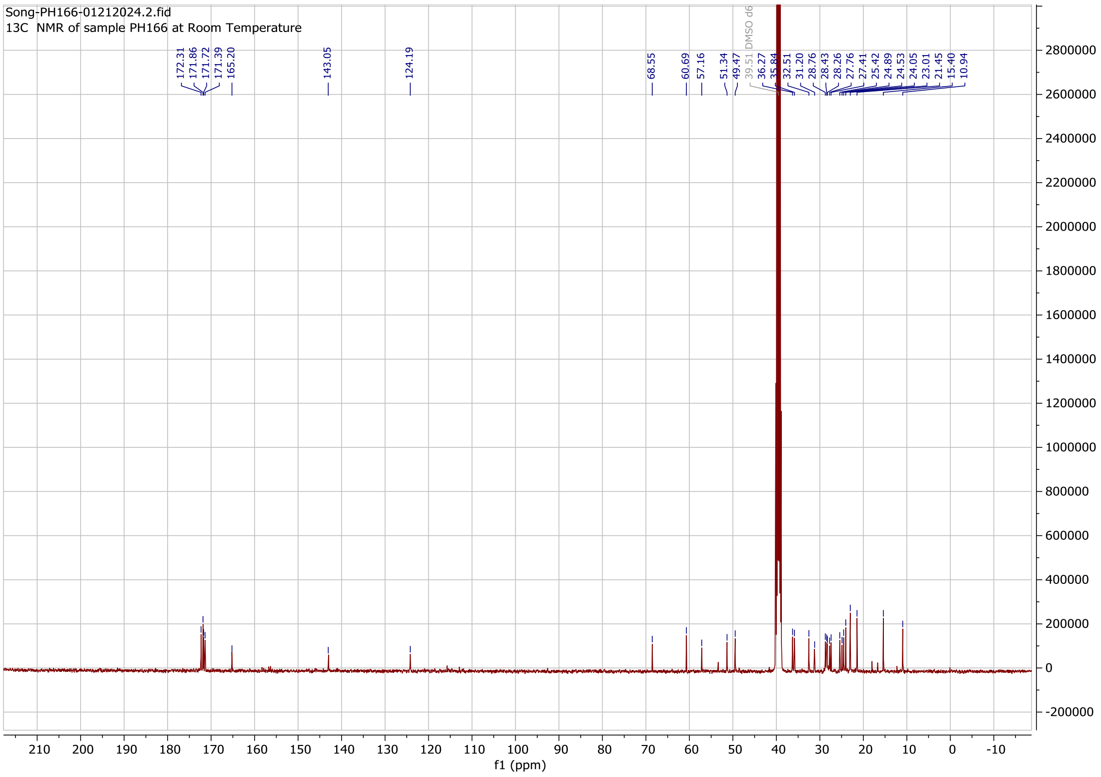

Figure S8. ^{13}C NMR spectrum of compound **3** (**hda-ILD-NH$_2$**, PH166) in DMSO-d6.

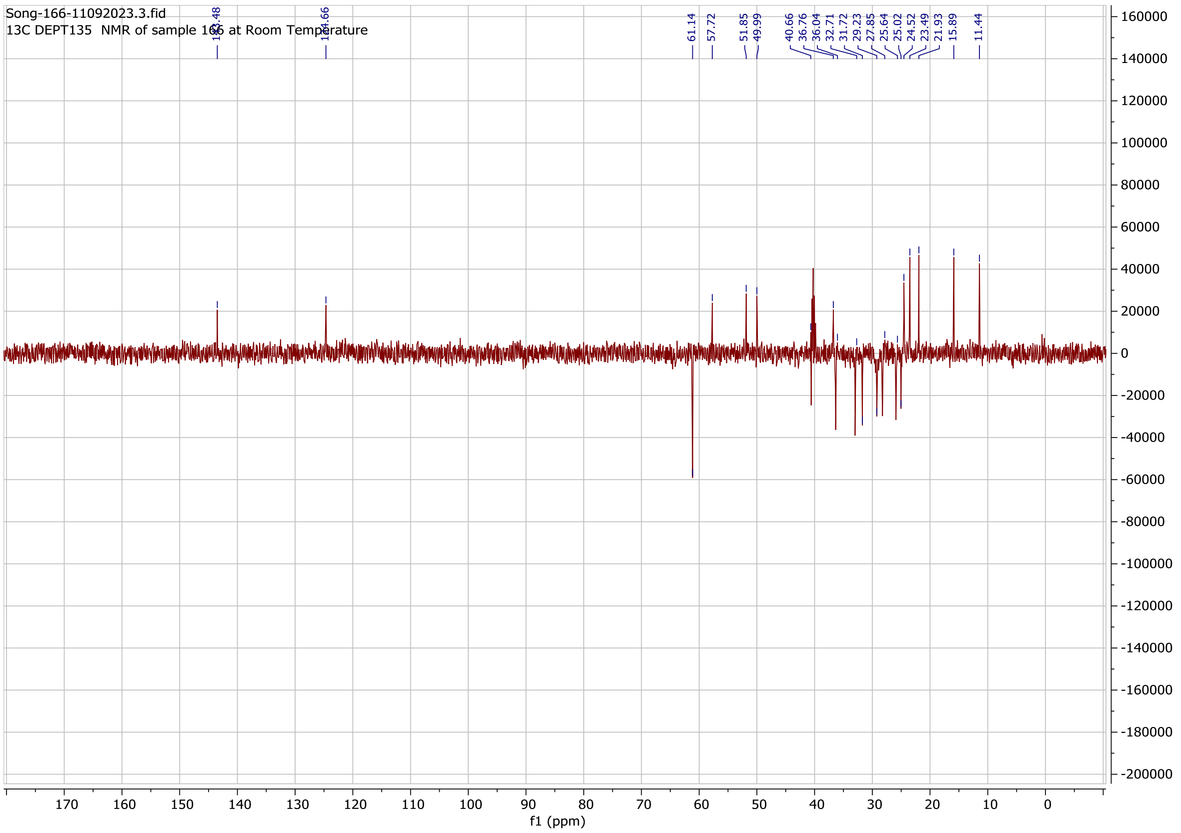

Figure S9. ^{13}C DEPT NMR spectrum of compound 3 (**hda-ILD-NH$_2$**, PH166) in DMSO-d6.

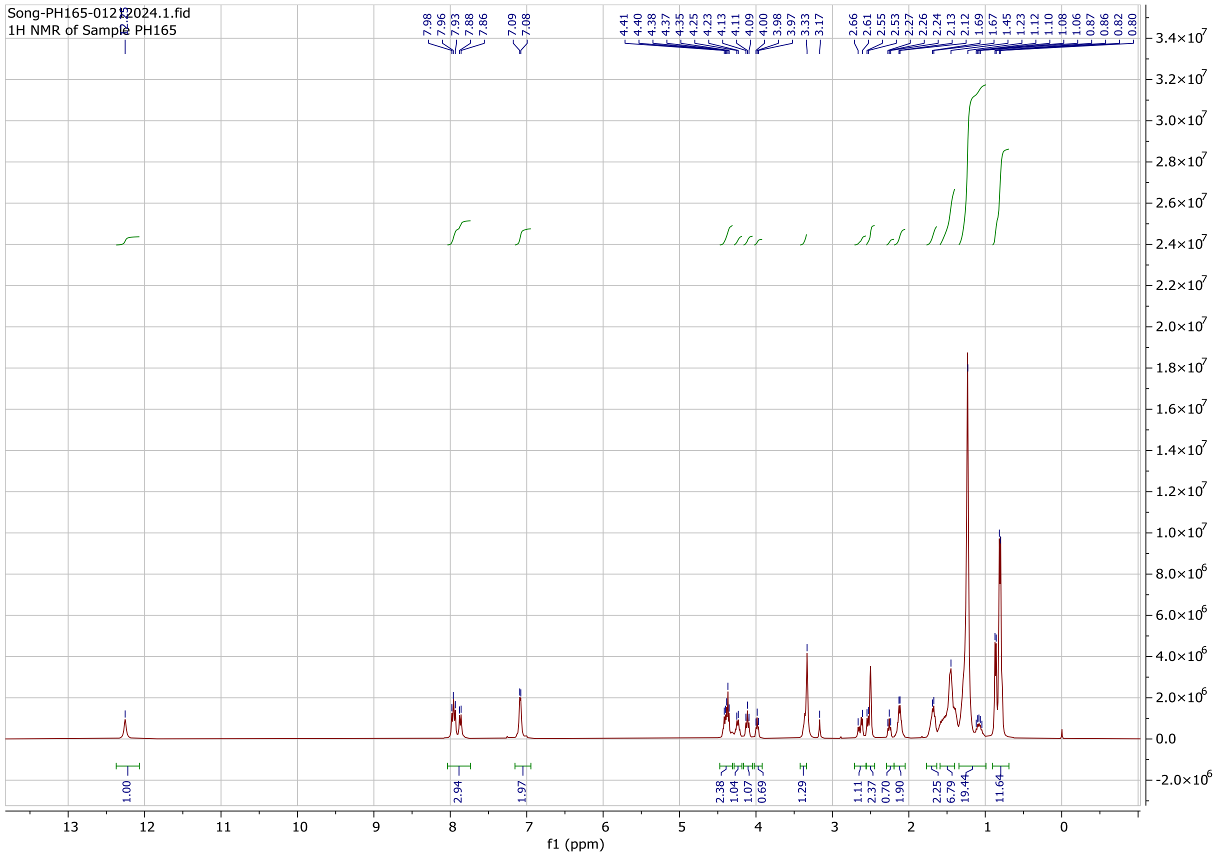

Figure S10. ^{1}H NMR spectrum of compound **4** (**12-hydroxyl dodecanoic acid-ILD-NH$_2$**, PH165) in DMSO-d6.

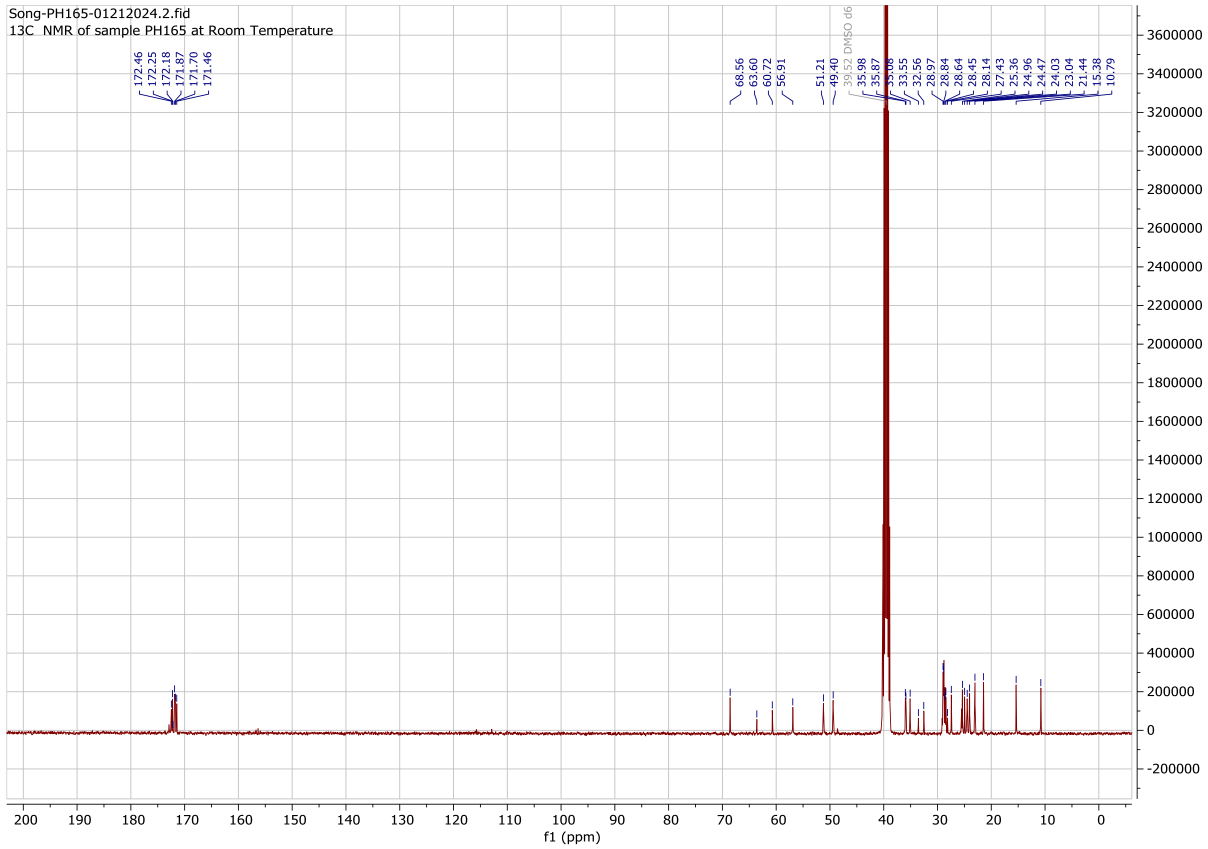

Figure S11. ^{13}C NMR spectrum of compound **4** (**12-hydroxyl dodecanoic acid-ILD-NH$_2$**, PH165) in DMSO-d6.

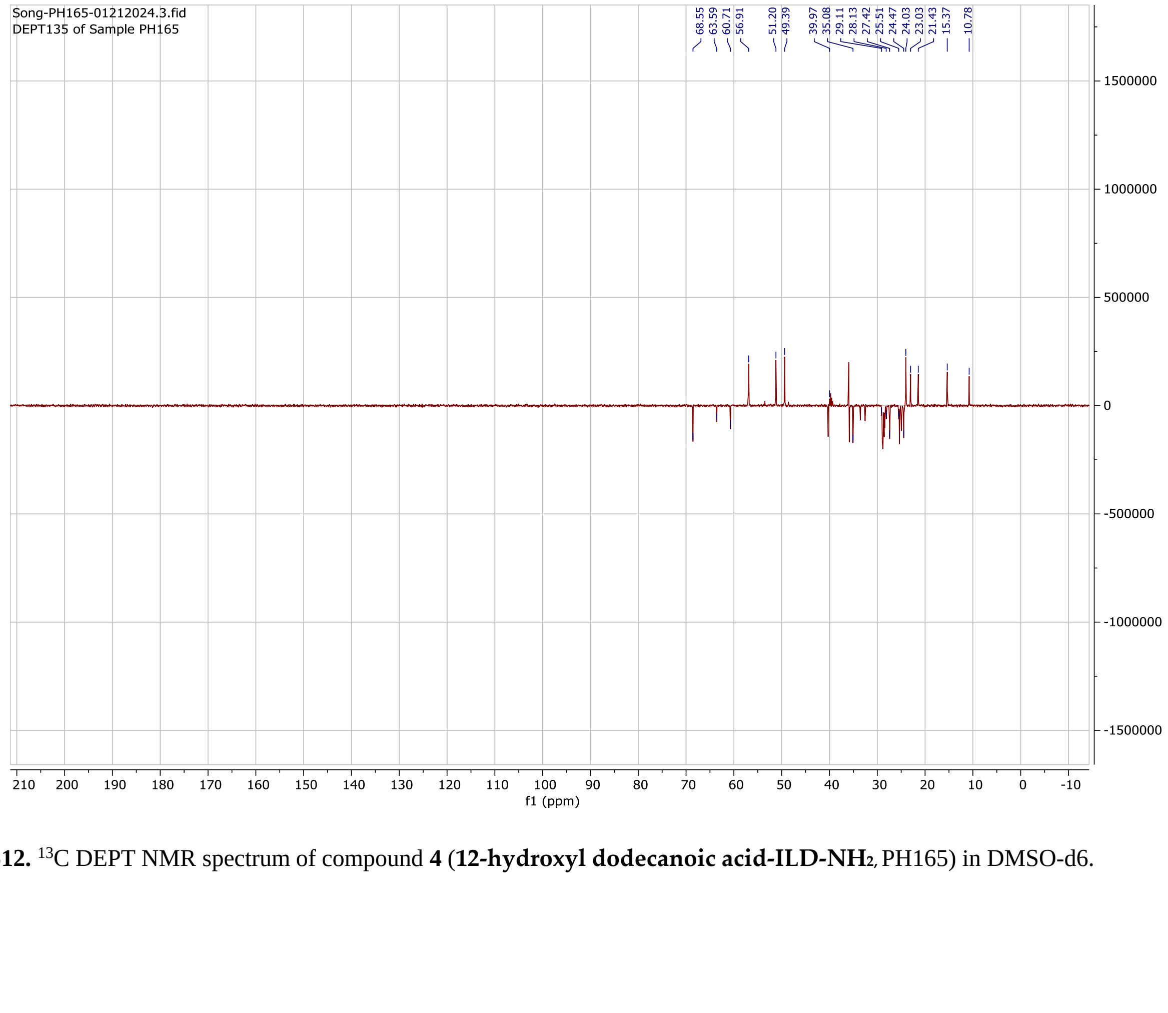

Figure S12. ^{13}C DEPT NMR spectrum of compound **4** (**12-hydroxyl dodecanoic acid-ILD-NH$_2$**, PH165) in DMSO-d6.

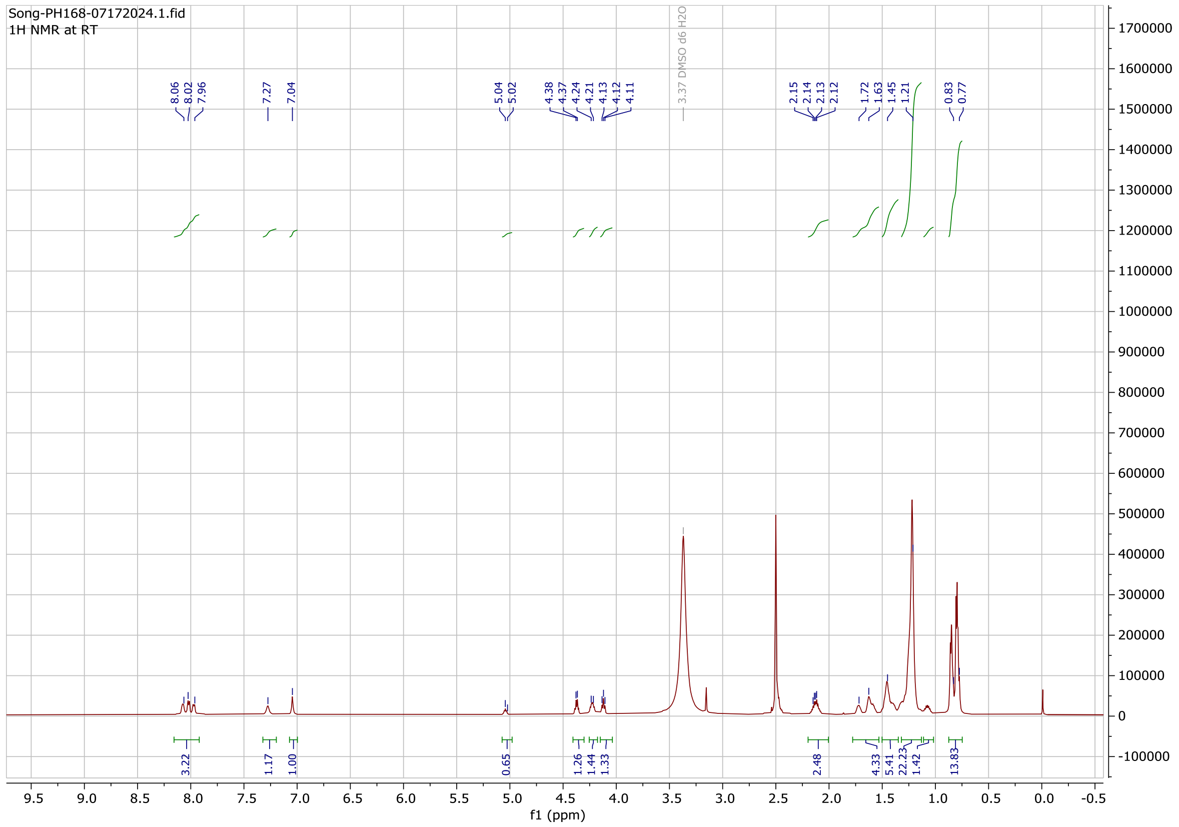

Figure S13. ^{1}H NMR spectrum of compound **5** (**12-hydroxyl octadecanoic acid-ILD-NH₂**, PH168) in DMSO-d6.

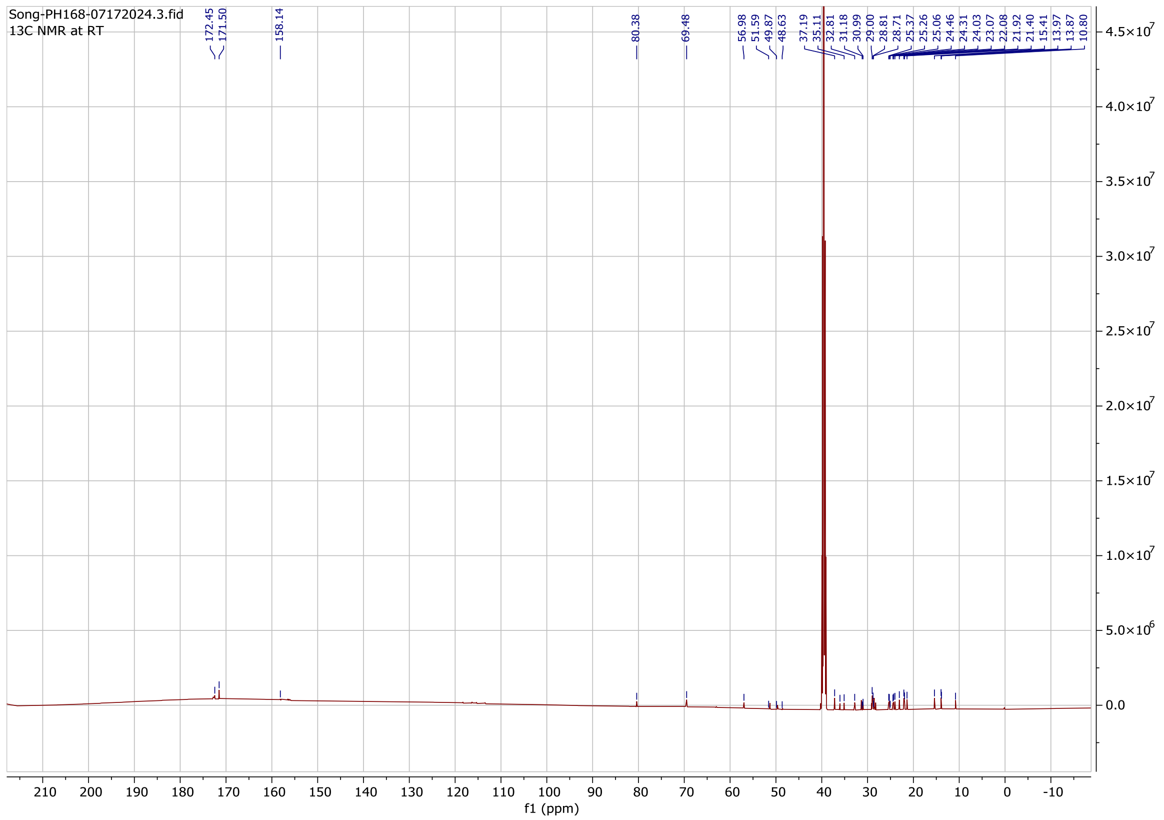

Figure S14. ^{13}C NMR spectrum of compound **5** (**12-hydroxyl octadecanoic acid-ILD-NH$_2$**, PH168) in DMSO-d6.

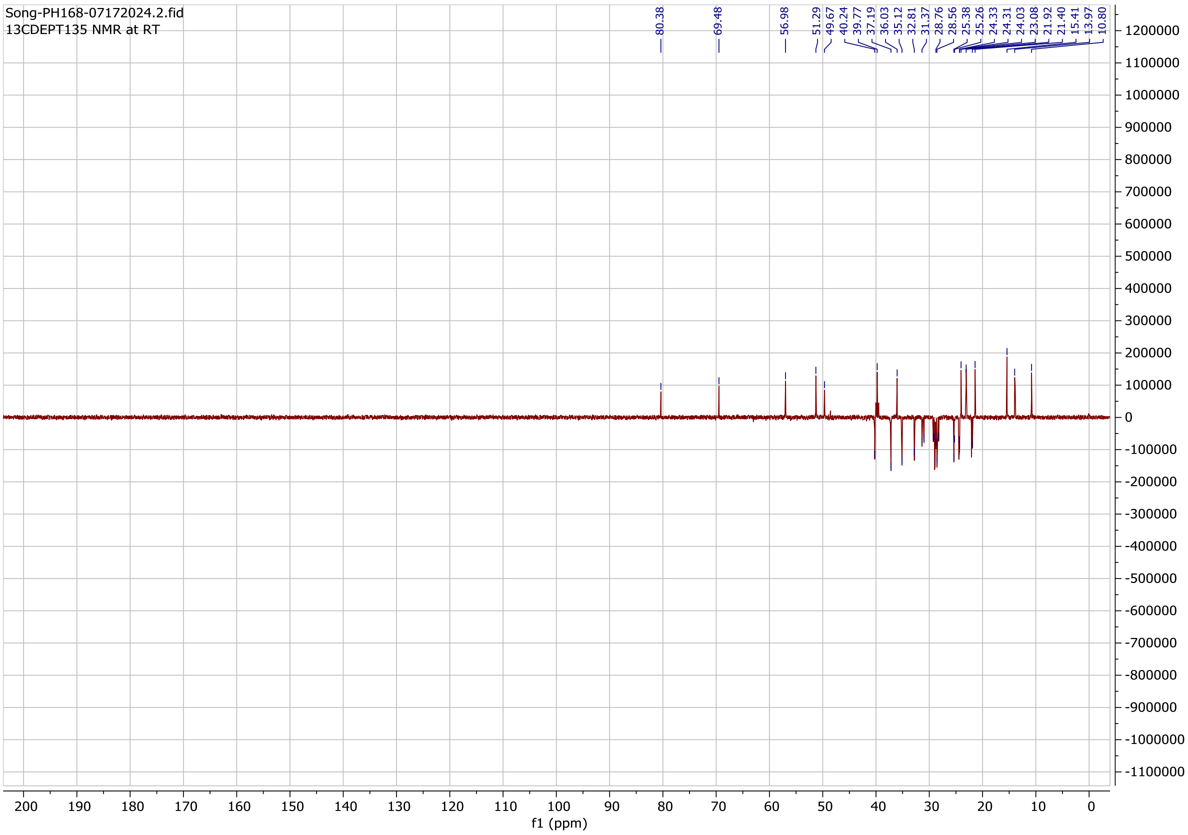

Figure S15. ^{13}C DEPT NMR spectrum of compound 5 (**12-hydroxyl octadecanoic acid-ILD-NH$_2$**, PH168) in DMSO-d6.

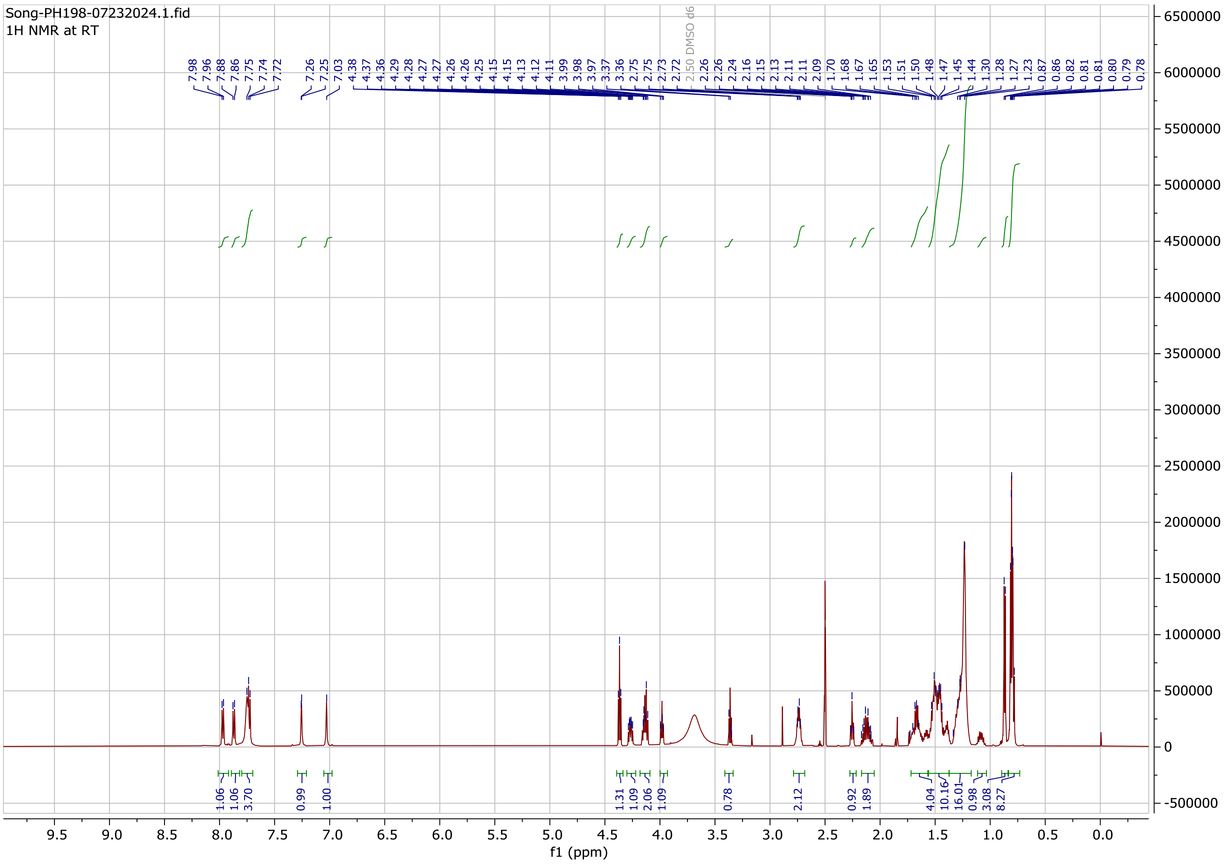

Figure S16. ^{1}H NMR spectrum of compound **6** (hdaa-ILK-NH$_2$, PH198) in DMSO-d6.

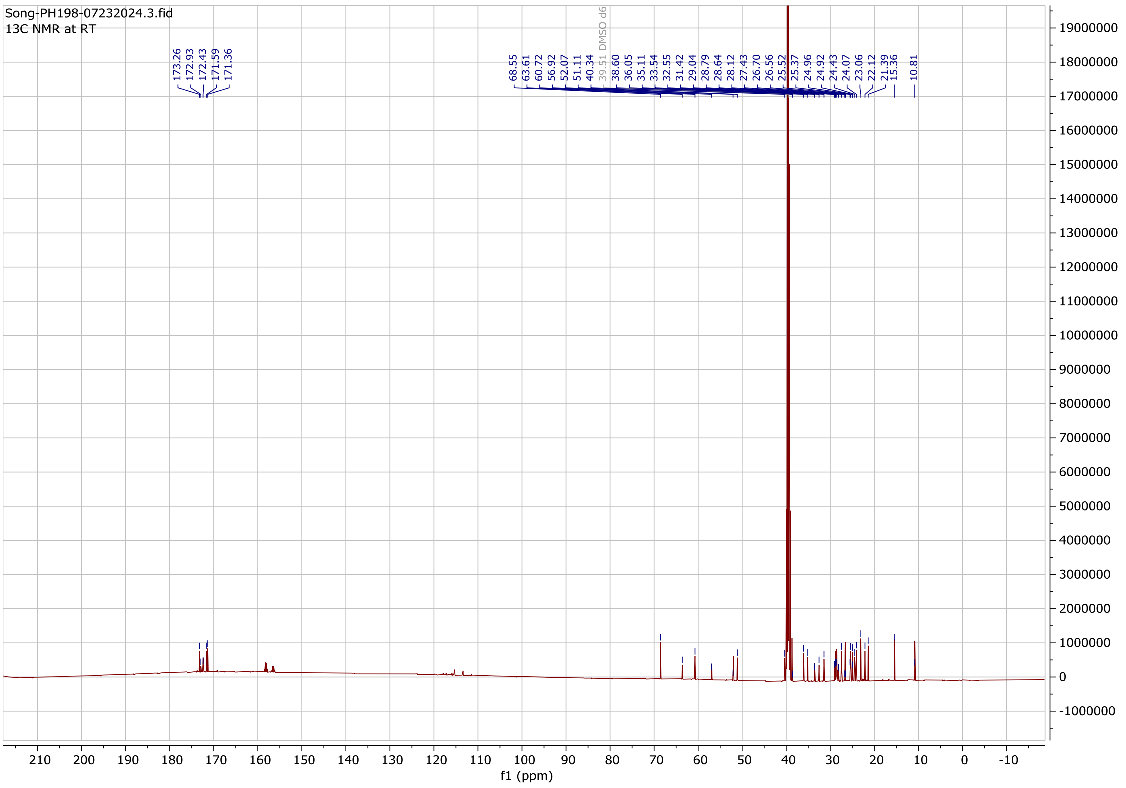

Figure S17. ^{13}C NMR spectrum of compound **6** (hdaa-ILK-NH_2, PH198) in DMSO-d6.

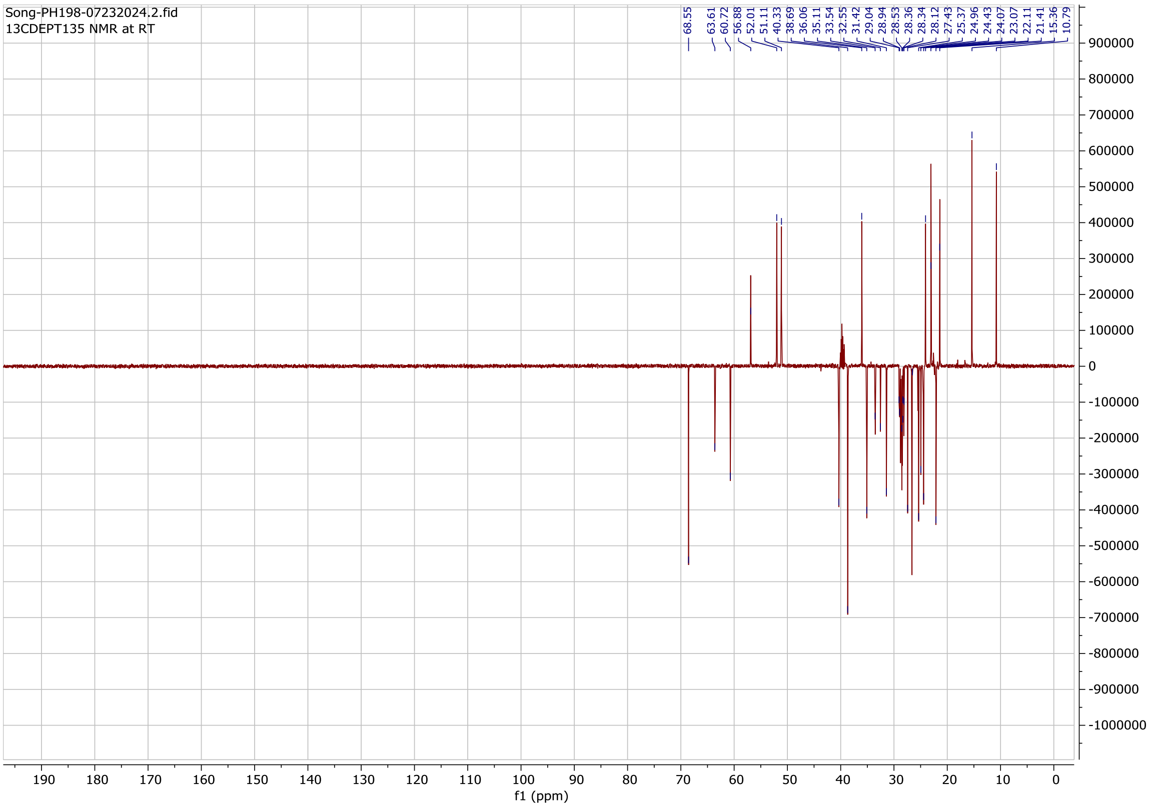

Figure S18. ^{13}C DEPT NMR spectrum of compound **6** (hdaa-ILK-NH_2, PH198) in DMSO-d6.

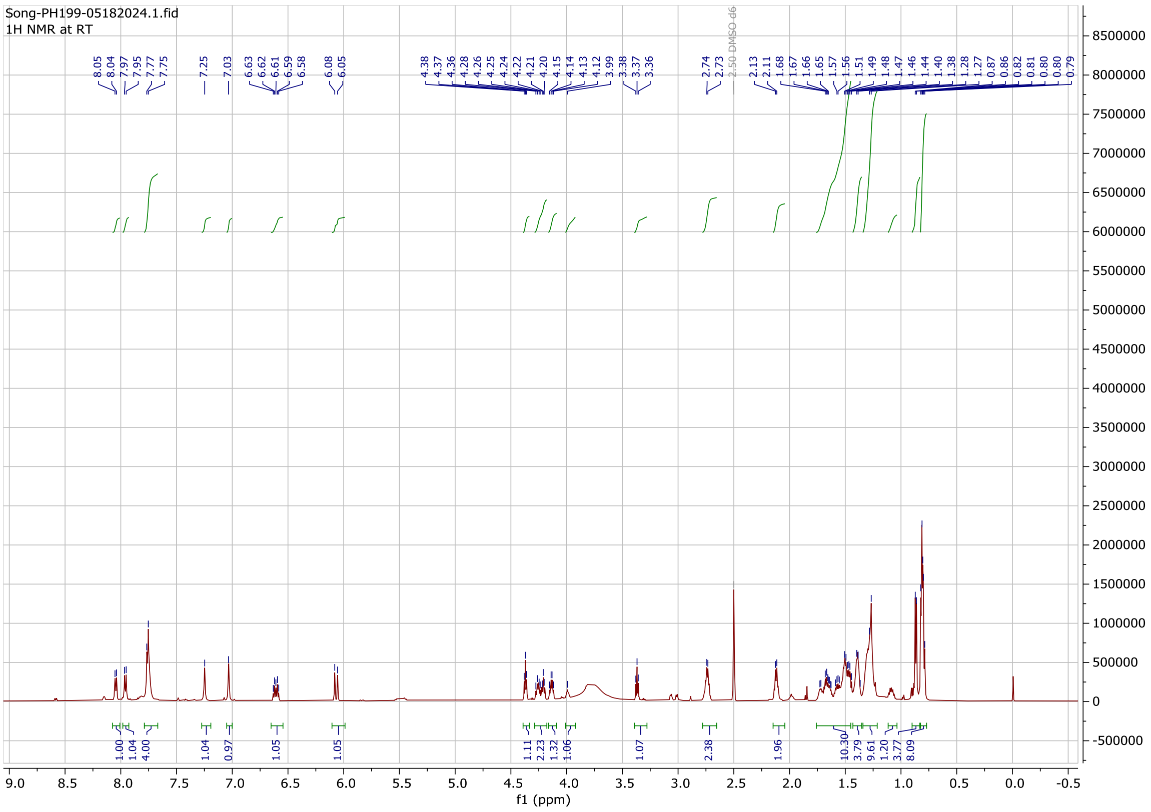

Figure S19. ^{1}H NMR spectrum of compound **7 (hda-ILK-NH$_2$,** PH199) in DMSO-d6.

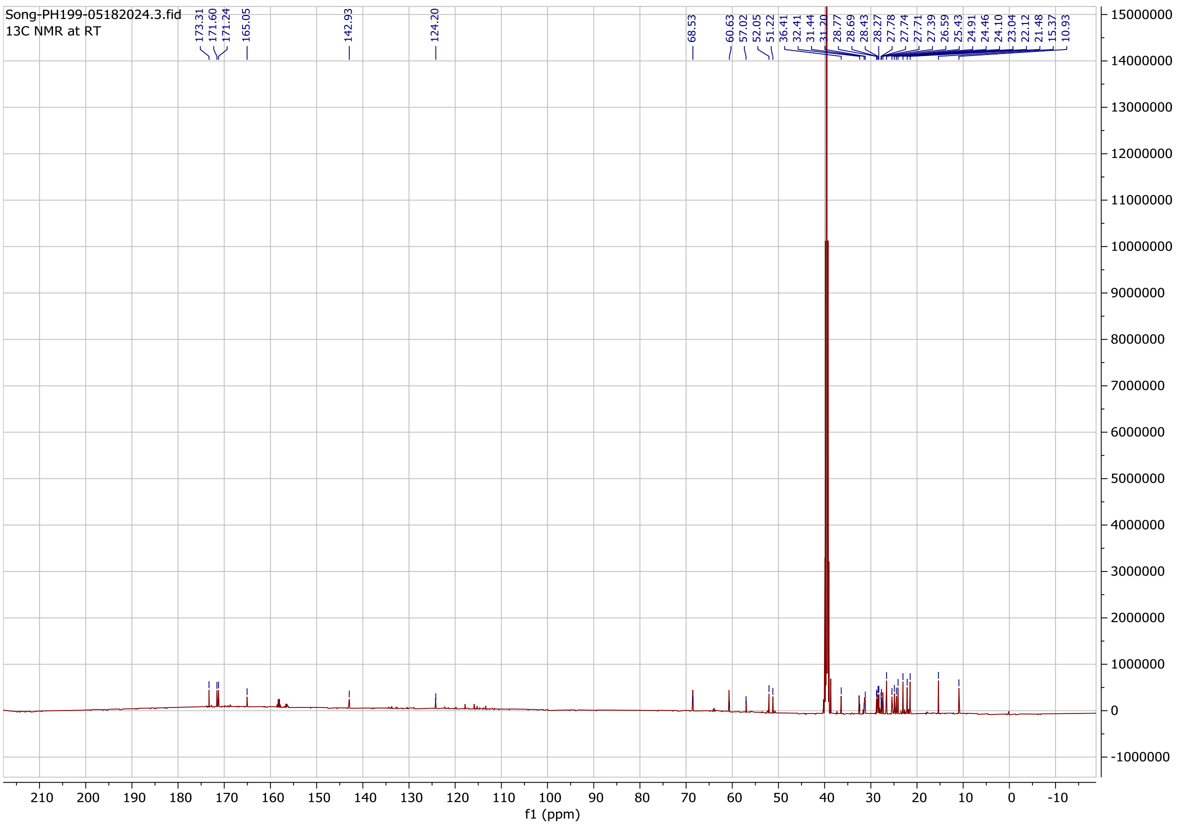

Figure S20. ^{13}C NMR spectrum of compound **7 (hda-ILK-NH$_2$,** PH199) in DMSO-d6.

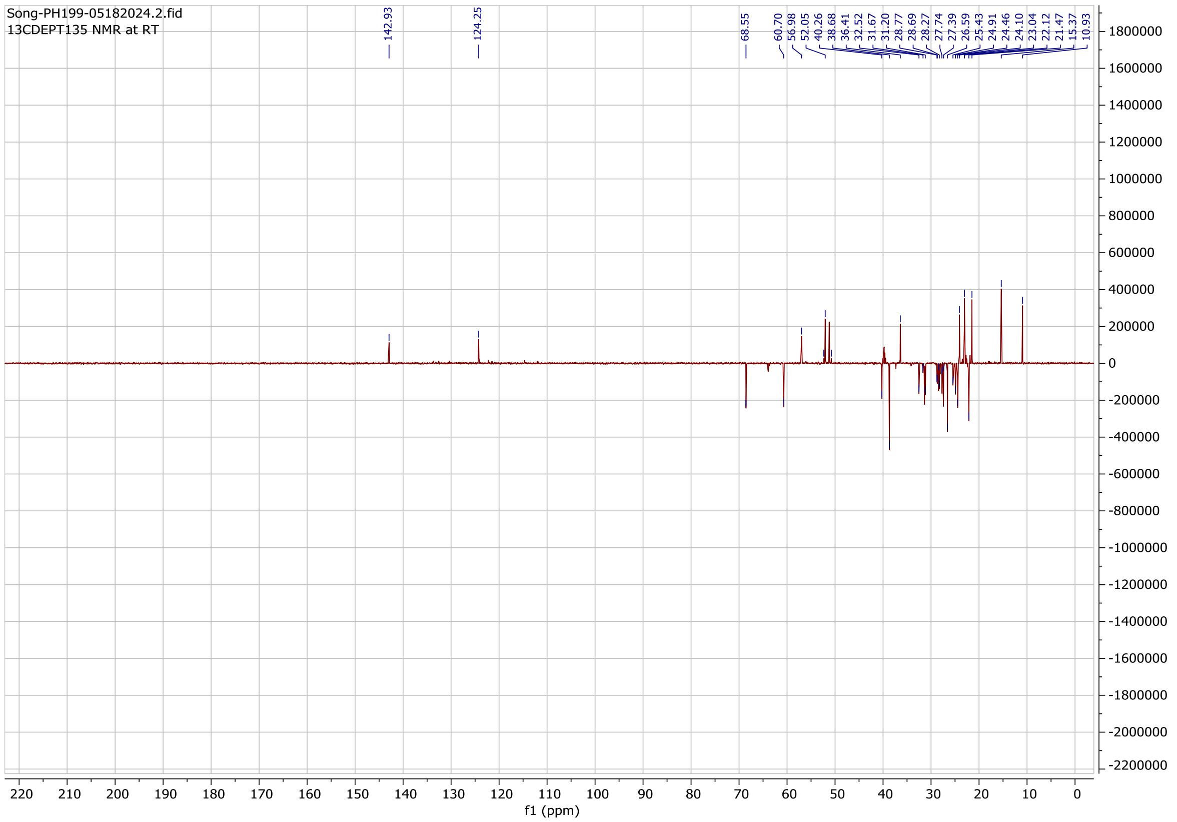

Figure S21. ^{13}C DEPT NMR spectrum of compound **7 (hda-ILK-NH$_2$,** PH199) in DMSO-d6.

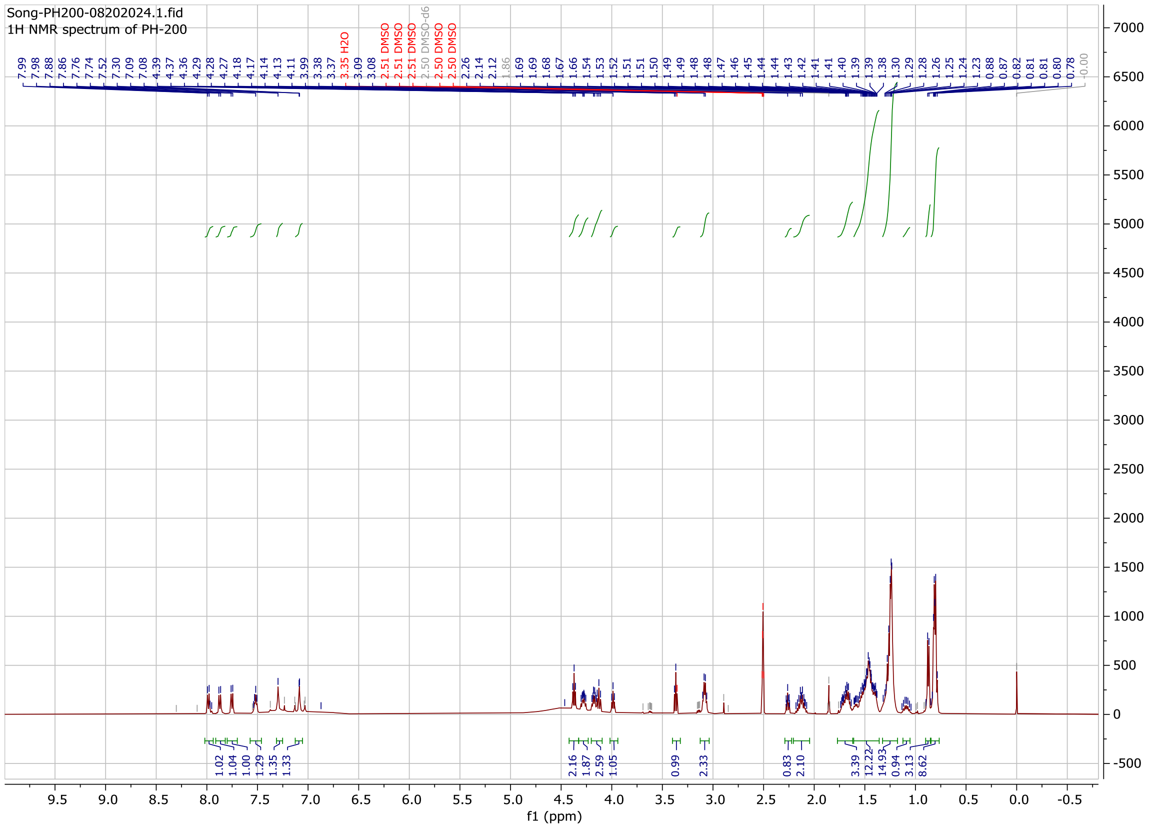

Figure S22. ^{1}H NMR spectrum of compound **8 (hdaa-ILR-NH$_2$,** PH200) in DMSO-d6.

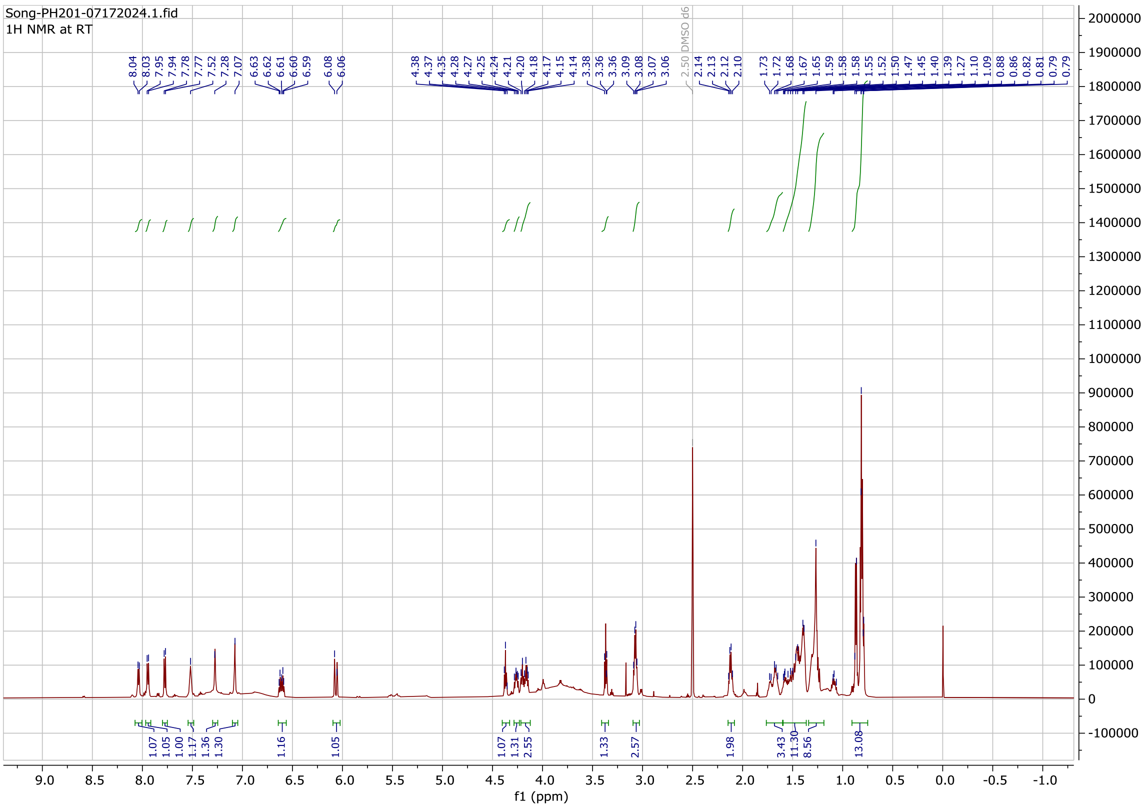

Figure S23. ^{1}H NMR spectrum of compound **9 (hda-ILR-NH$_2$,** PH201) in DMSO-d6.

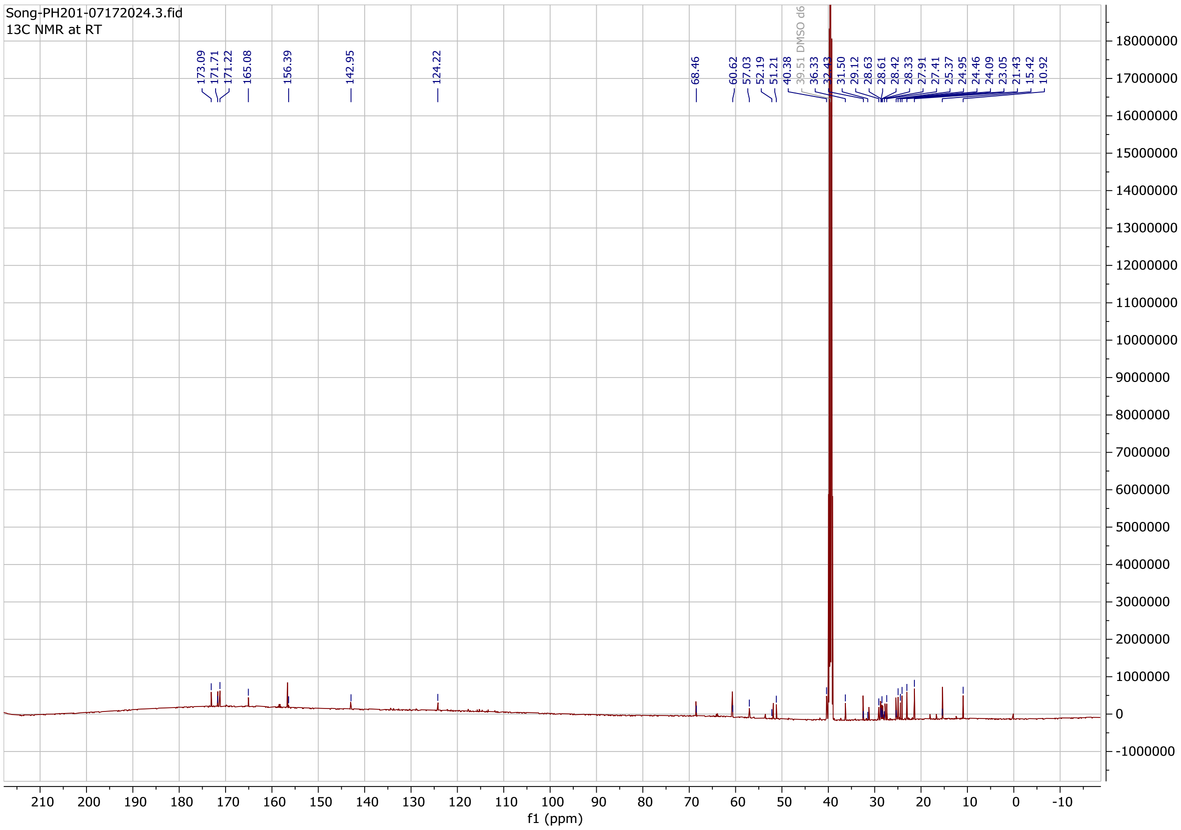

Figure S24. ^{13}C NMR spectrum of compound **9 (hda-ILR-NH$_2$,** PH201) in DMSO-d6.

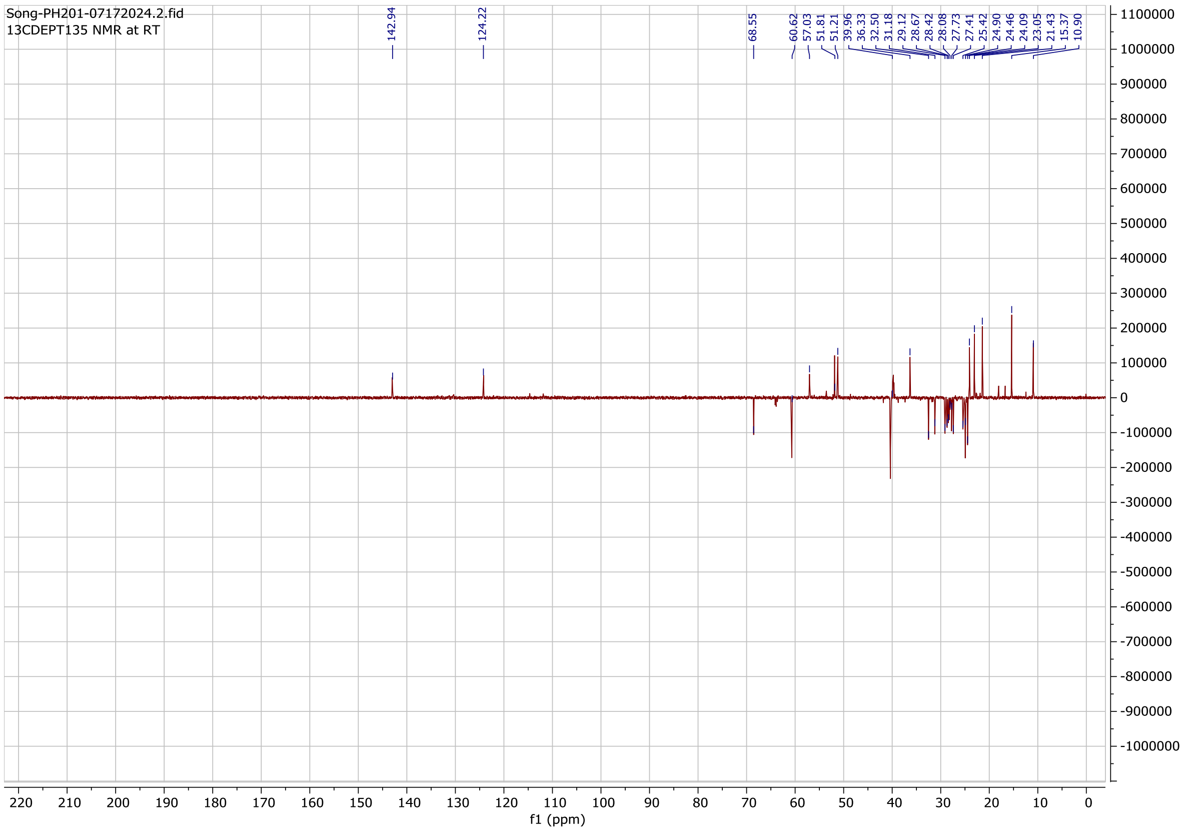

Figure S25. ^{13}C DEPT NMR spectrum of compound **9 (hda-ILR-NH2,** PH201) in DMSO-d6.

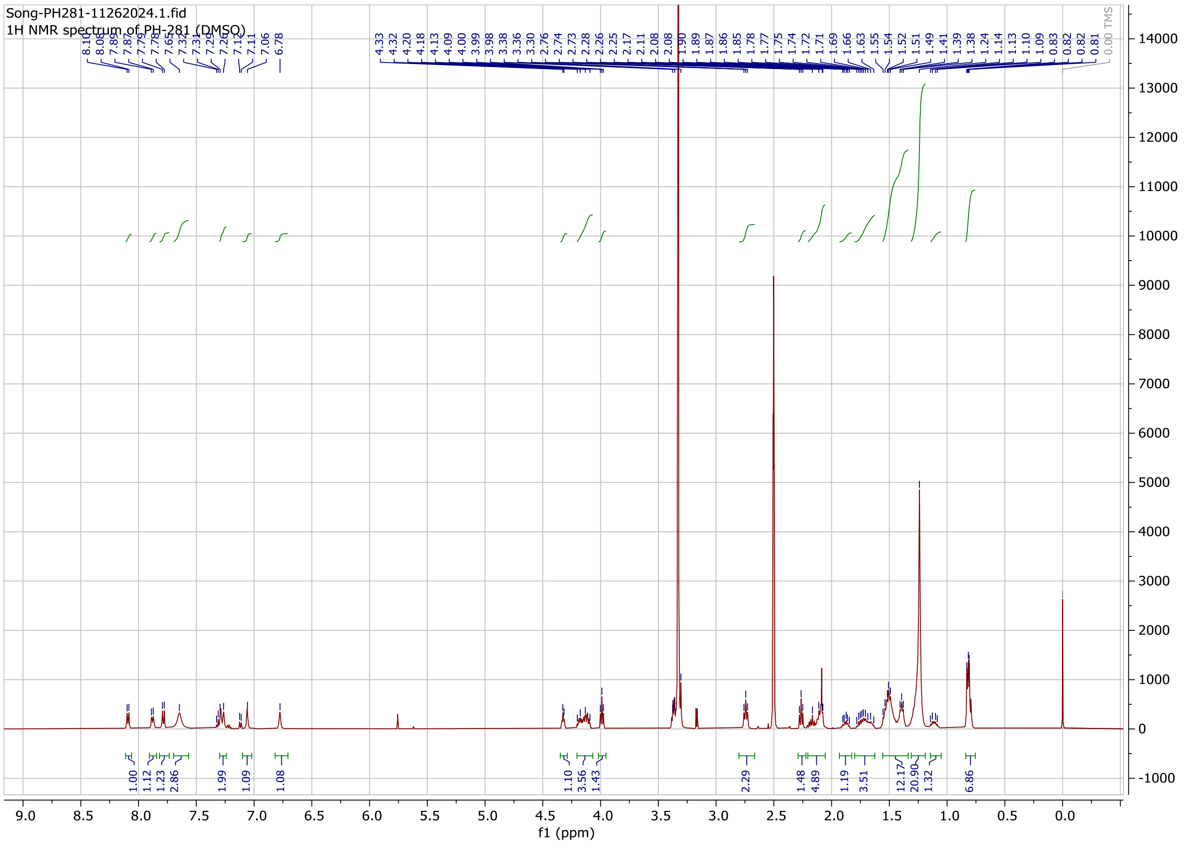

CV

Figure S26. ^{1}H NMR spectrum of compound **10 (**hdaa-IQK-NH2, PH281) in DMSO-d6.

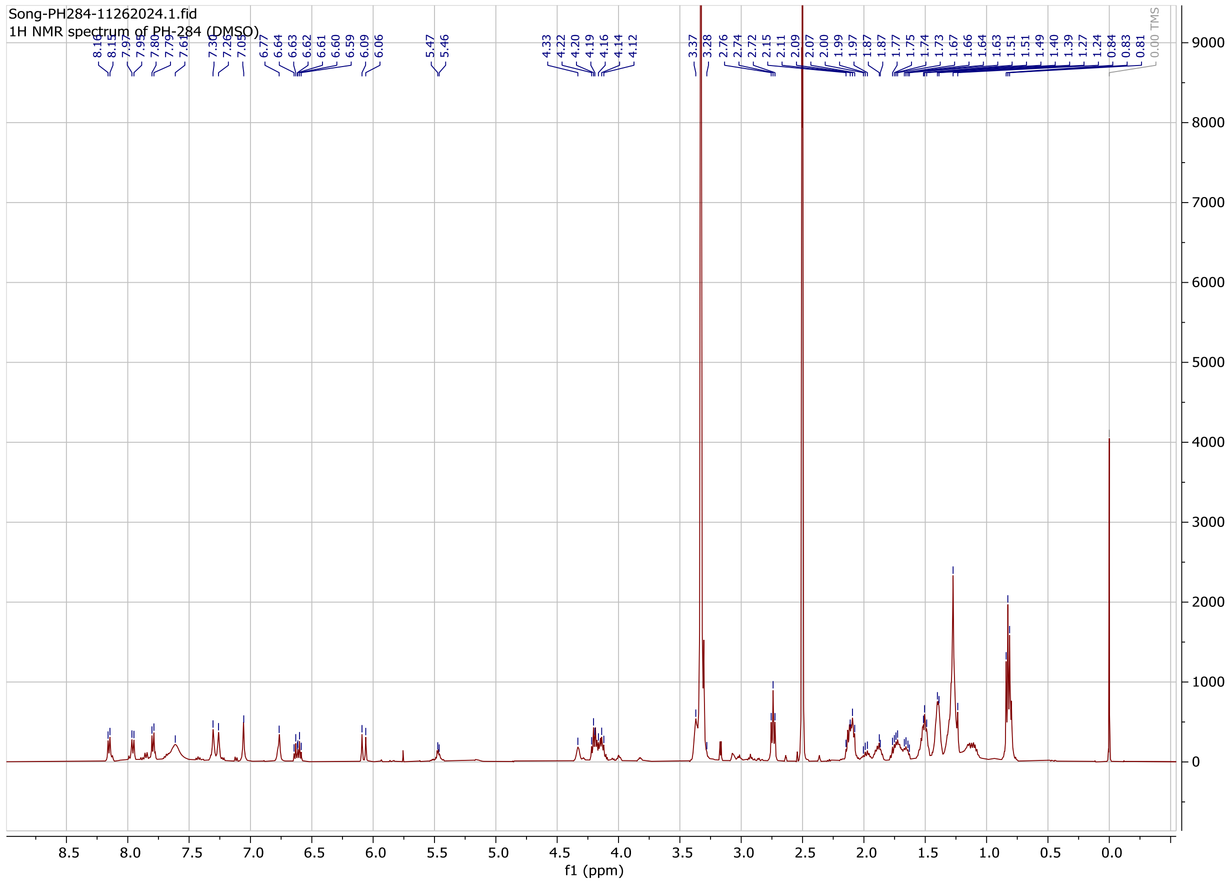

Figure S27. ^{1}H NMR spectrum of compound **11 (**hda-IQK-NH_2, PH284) in DMSO-d6.
